# Tensorial Properties via the Neuroevolution Potential Framework: Fast Simulation of Infrared and Raman Spectra

**DOI:** 10.1021/acs.jctc.3c01343

**Published:** 2024-04-04

**Authors:** Nan Xu, Petter Rosander, Christian Schäfer, Eric Lindgren, Nicklas Österbacka, Mandi Fang, Wei Chen, Yi He, Zheyong Fan, Paul Erhart

**Affiliations:** † Institute of Zhejiang University-Quzhou, Quzhou 324000, P. R. China; ‡ College of Chemical and Biological Engineering, Zhejiang University, Hangzhou 310058, P. R. China; ¶ Department of Physics, Chalmers University of Technology, SE-41296 Gothenburg, Sweden; § State Key Laboratory of Multiphase Complex Systems, Institute of Process Engineering, Chinese Academy of Sciences, Beijing 100190, P. R. China; ∥ Department of Chemical Engineering, University of Washington, Seattle, Washington 98195, United States; ⊥ College of Physical Science and Technology, Bohai University, Jinzhou 121013, P. R. China

## Abstract

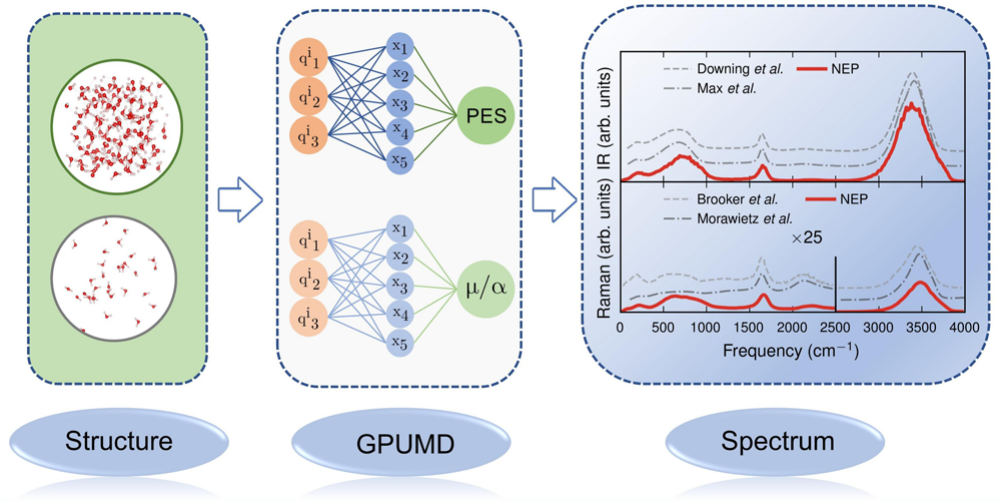

Infrared and Raman spectroscopy are widely used for the characterization of gases, liquids, and solids, as the spectra contain a wealth of information concerning, in particular, the dynamics of these systems. Atomic scale simulations can be used to predict such spectra but are often severely limited due to high computational cost or the need for strong approximations that limit the application range and reliability. Here, we introduce a machine learning (ML) accelerated approach that addresses these shortcomings and provides a significant performance boost in terms of data and computational efficiency compared with earlier ML schemes. To this end, we generalize the neuroevolution potential approach to enable the prediction of rank one and two tensors to obtain the tensorial neuroevolution potential (TNEP) scheme. We apply the resulting framework to construct models for the dipole moment, polarizability, and susceptibility of molecules, liquids, and solids and show that our approach compares favorably with several ML models from the literature with respect to accuracy and computational efficiency. Finally, we demonstrate the application of the TNEP approach to the prediction of infrared and Raman spectra of liquid water, a molecule (PTAF^–^), and a prototypical perovskite with strong anharmonicity (BaZrO_3_). The TNEP approach is implemented in the free and open source software package gpumd, which makes this methodology readily available to the scientific community.

## 1 Introduction

Infrared (IR) and Raman spectroscopy are widely used techniques for the nondestructive characterization of the dynamics and to some extent chemistry of materials spanning the entire range from the gas phase to condensed matter.^1−3^ Over the years, various theoretical approaches have been developed for simulating IR and Raman spectra, including in particular methods based on *ab initio* molecular dynamics (MD) simulations.^4−8^ While these approaches are capable of reproducing experimental IR and Raman spectra of gases, liquids, and solids,^5,7−9^ they are severely limited with respect to the system sizes and time scales attainable for two main reasons:^5,10^ First, *ab initio* MD simulations rely on computationally demanding electronic structure calculations that scale strongly with system size in order to obtain energy and forces at every time step. Second, similarly expensive calculations of dipole moment (**μ**), polarizability (**α**), or electric susceptibility (**χ**) are required for at least many thousands of configurations to achieve numerical convergence of the underlying correlation functions.^5^

MD simulations can be accelerated by using classical force fields^11−13^ or empirical interatomic potentials,^14,15^ which approximate the potential energy surface (PES) with physically motivated yet constrained functions and few fitted parameters. The accuracy of such approaches for general materials is, however, often limited, negatively affecting the prediction of IR and Raman spectra.^16^ Machine learning (ML) potentials are well suited to address this challenge as they bridge between the accuracy of quantum mechanical methods and the computational efficiency of classical force fields or empirical interatomic potentials.^17−21^ The power of this approach, in particular for capturing vibrational properties of materials, has been shown repeatedly, see, e.g., refs.^22−26^

The calculation of **μ**, **α**, or **χ** can be accelerated using parametric models in a similar fashion. Considering only static charges, the dipole moment is given by **μ** = ∑_*i*=1_^*N*^*Q*_*i*_***r***_*i*_, where *Q*_*i*_ and ***r***_*i*_ are the charge and position of atom *i*. Many classical force fields^11−13^ assign fixed charges to atoms and thereby provide a convenient approach for calculating **μ**. Such fixed-charge models neglect, however, polarization effects, which can lead to large errors.^27^ While this situation can in principle be ameliorated by fluctuating-charge models,^28,29^ the latter tend to lack robustness and can be difficult to generalize.^10,30^

Both **α** and **χ** describe the dielectric response to an applied electric field. For **α** or **χ**, the bond polarizability model is one of the most frequently used parametric ones and has for example been applied to alkanes^31,32^ and zeolites^33^ as well as carbon nanotubes.^34^ However, this simple model often suffers from unsatisfactory transferability when used in different environments.^35^ POLI2VS^36^ and MB-pol^37^ are two other parametric models that can be used for predicting **μ** and **α** but are limited to molecular systems such as water.^10^

The successful applications of ML potentials have inspired the development of ML dipole, polarizability, and susceptibility models.^22,38−41^ For **μ**, a rank-1 tensor, both partial-charge and the partial-dipole ML models have been developed.^30^ The objective of the partial-charge models is to assign proper partial charges for atoms in order to fit the total dipole moment.^22,30,42^ Here, one concern is the balance between the fitting quality of **μ** and the reproducibility of total charges.^22,30^ By contrast partial-dipole models such as symmetry-adapted Gaussian process regression (SA-GPR),^38^ tensorial embedded-atom neural network (T-EANN),^39^ and deep potential (DP)^40^ treat **μ** as a sum of vectors^30,38^ that can be determined from atom-centered chemical environments.

While this approach works for **μ**, which is a rank-1 tensor, it does not transfer to the construction of ML models for **α** or **χ**, which are rank-2 tensors. This has motivated the pioneering development of the SA-GPR method for tensorial properties^38^ as well as later the T-EANN^39,43^ and DP models.^10^

The combination of ML potentials with ML models for **μ**, **α**, or **χ** enables the simulations of IR and Raman spectra. This approach has been used to predict, e.g., the IR spectra of methanol, *n*-alkanes, and a peptide,^22^ IR and Raman spectra of liquid water,^10,20,39,44^ or the Raman spectra of various solid materials.^45^ While these earlier studies have established the usefulness of ML models for predicting IR and Raman spectra, there is still ample room for improvement of current models for **μ**, **α**, or **χ** in terms of computational and data efficiency^30,39^ as well as the accessibility of these techniques in order to lower the threshold for the widespread adoption of such approaches.

**Figure 1:**
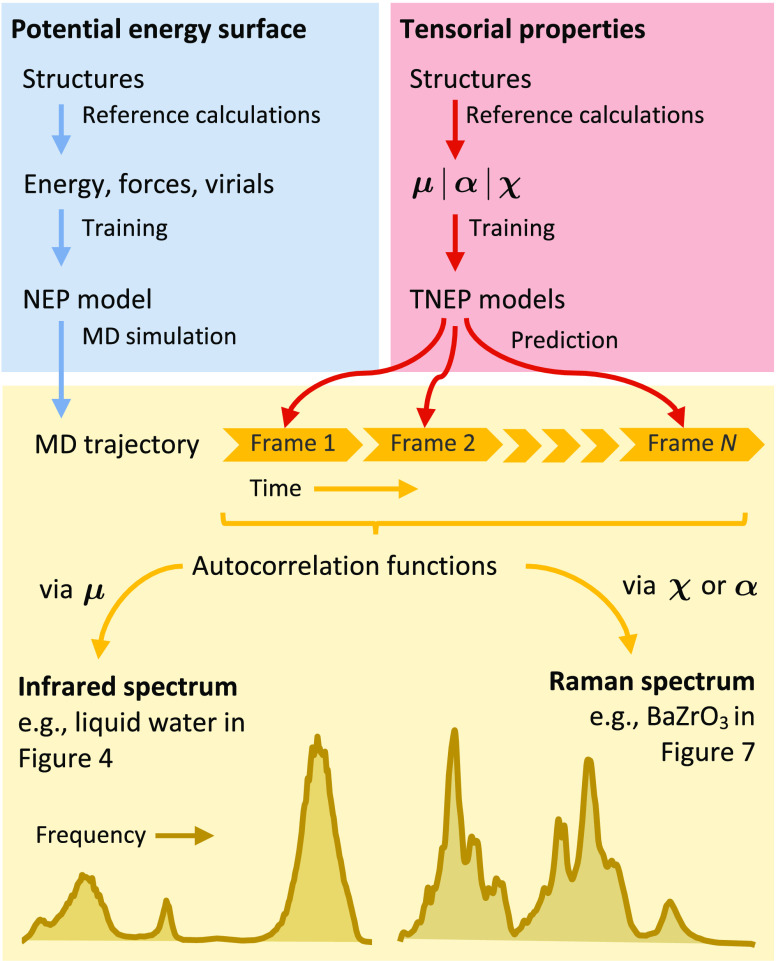
Workflow for simulations of IR and Raman spectra using NEP models for the PES and TNEP models for the dipole moment **μ**, the polarizability **α**, or the susceptibility **χ**.

This situation motivates the present work, in which we introduce accurate as well as computationally and data efficient ML models for rank-1 and rank-2 tensors based on the NEP framework.^21,46,47^ We demonstrate the efficacy and efficiency of the resulting TNEP approach by training models for **μ**, **α**, and **χ** and combining these with models for the PES to predict IR and Raman spectra for a molecule (PTAF^–^), a liquid (water), and a solid (BaZrO_3_; Figure 1). We make this methodology available via the gpumd package,^47^ enabling comprehensive simulations of high-quality IR and Raman spectra with limited user effort.

## 2 Methodology

### 2.1 NEP Models for the PES

Since the ML models for **μ** and **α** that we introduce below are based on the NEP framework for modeling PESs,^21,46,47^ we first provide a brief review of the latter. Originally NEPs are ML potentials that model the high-dimensional PES of finite or extended systems, in the spirit of the neural network potential model proposed by Behler and Parrinello.^48^ In this formalism, the total energy of the system is given by the sum of atomic site energies *U* = ∑_*i*_*U*_*i*_. The site energy *U*_*i*_ for a given atom *i* depends on the local environment of the atom, which is represented by an abstract vector *q*_*i*_^ν^ with a number of components indexed by ν. The function mapping from the descriptor to the site energy is represented by a feed-forward neural network (also known as a multilayer perceptron) with typically a single hidden layer. The input layer of the neural network is thus the descriptor vector, and the output layer consists of a single node whose value is the site energy *U*_*i*_ of the considered atom *i*, which can be formally expressed as
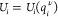
1

From the energy, we can derive the rank-2 virial tensor that serves as the foundation for the dipole and polarizability models developed in the present work. For a given structure with *N* atoms, the virial tensor can be expressed as^47^
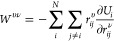
2where *r*_*ij*_^υ^ is the υ-component of the vector ***r***_*ij*_ ≡ ***r***_*j*_ – ***r***_*i*_, and ***r***_*i*_ is the position of atom *i*. We refer to the term ∂*U*_*i*_/∂*r*_*ij*_^ν^ as the partial force, explicit expressions for which have been presented in the original works developing the NEP approach.^21,47^

### 2.2 TNEP Rank-1 Tensor Models

To develop an ML model for predicting **μ**, we first note that it is a rank-1 tensor commonly expressed as a vector, in contrast to the energy, which is a rank-0 tensor (i.e., a scalar). The partial force in eq 2 is a vector, but the summation of it over the whole structure would be zero as a result of Newton’s third law. To obtain a vector representation that does not vanish for a general structure, we note that the quantity defined in eq 2 is a rank-2 tensor that can adopt both positive and negative values (as it is the virial tensor in the context of PES models). We can thus obtain an expression for a vector quantity by contracting this rank-2 tensor with a vector. A natural choice for the vector to be contracted is ***r***_*ij*_, which yields the following expression for rank-1 tensors such as the dipole moment
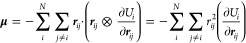
3where *r*_*ij*_^2^ = ***r***_*ij*_·***r***_*ij*_ is the distance squared between atoms *i* and *j*. We note that *U*_*i*_ here should have the dimension of charge instead of energy. Crucially this goes to show that the NEP formalism for PESs can be directly used to construct an ML model for rank-1 tensors such as the dipole moment. Below we refer to eq 3 as the TNEP dipole model.

### 2.3 TNEP Rank-2 Tensor Models

To develop ML models for predicting **α** or **χ**, we first note that these are rank-2 tensors. Clearly, the quantity defined in eq 2 is an ideal candidate. However, using only eq 2 to represent **α** or **χ** does not lead to high regression accuracy because the diagonal terms of **α** or **χ** are usually much larger than the off-diagonal ones. We therefore represent **α** (and equivalently **χ**) as a combination of eqs 1 and 2 as follows
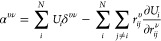
4where δ^*υν*^ is the Kronecker delta. Note that both the first and second terms on the right-hand side contribute to the diagonal elements of α^*υν*^, but only the second term contributes to the off-diagonal elements. *U*_*i*_ here has the dimension of polarizability instead of energy, yet the entire NEP formalism can be reused. Below we refer to eq 4 as the TNEP polarizability or susceptibility model.

### 2.4 Loss Functions

The NEP approach is named after the underlying ML model (a neural network) and the separable natural evolution strategy used as the training algorithm.^49^ The latter is a principled real-valued black-box optimization method that is very well suited for training the weight and bias parameters in the neural network, of which there are typically a few thousand. The optimization is driven by the minimization of a loss function that is given by the weighted sum of the root-mean-square error (RMSE) of physical quantities as well as  and  regularization terms. For the construction of PES models, the physical quantities included in the loss function are the energies, forces, and virial tensors of the structures in the training set
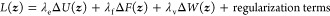
5where *ΔU*(***z***), *ΔF*(***z***), and *ΔW*(***z***) are the RMSEs of energies, forces, and virials calculated using a set of trainable parameters ***z***, and λ_e_, λ_f_, and λ_v_ are the corresponding relative weights. Explicit expressions for the regularization terms can be found in ref (47). For the construction of dipole TNEP models, the loss function is defined in terms of the RMSE of the dipole *Δμ*(***z***)
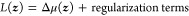
6For the construction of polarizability TNEP models, the loss function is defined in terms of the RMSE of the polarizability *Δα*(***z***)
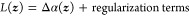
7

### 2.5 Dielectric Response

It is instructive to recall some relations that describe the response of finite systems (such as molecules) and extended systems (such as solids and liquids) to an applied electric field.

If a molecule is subjected to an electric field ***E***, the resulting displacement of nuclei and electrons induces a dipole, which is given by^50^
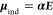
where **α** is the *molecular polarizability*.

For an extended system such as a solid or a liquid, one considers equivalently the dipole moment per unit volume, i.e., the polarization
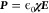
where **χ** is the *electric susceptibility*. In the context of bulk liquids, the latter has also been referred to as the bulk polarizability. For clarity in the following, we use the term polarizability only to refer to molecular polarizability. There are different conventions for expressing **μ**, **α**, and **χ** leading to different units (Sect. S7). Here, we use e·bohr for **μ** and bohr^3^ for **α**, whereas **χ** is unitless.

We note that under certain conditions, one can approximately connect the molecular polarizability and the electric susceptibility via the Clausius-Mossotti relation, which is based on a mean-field treatment of local field effects (see Sect. S8 in the Supporting Information).

### 2.6 The IR Intensity

The IR absorption cross section is given by^50^
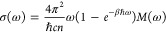
8where *n* is the refractive index of the material, *c* is the speed of light, β = 1/*k*_B_*T*, and *M*(ω) is the absorption line shape given by the Fourier transform of the autocorrelation function (ACF) of the (total) dipole moment **μ**
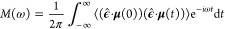
where ⟨···⟩ indicates the average over time origins, and **ϵ̂** is the polarization of the light.^50^ For an isotropic sample, the time correlation should be averaged over the three directions, i.e., the line shape reduces to one-third of the trace of the dipole time correlation. Since the line shape is sampled classically, we make a classical approximation for the prefactor by expanding the Boltzmann factor to first order, which gives
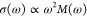
9

### 2.7 The Raman Intensity

The differential Raman cross-section for Stokes scattering is given by^50−52^
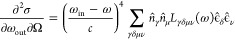
10where *n̂* is the polarization of observed light, **ϵ̂** is the polarization of the incoming light, and Ω is a solid angle. Here, it is assumed that the frequency of the incoming light ω_in_ is significantly larger than the Raman shift ω and significantly smaller than the band gap, i.e., far from any electronic excitations. *L*(ω) is the Raman line shape given by the Fourier transform of the time-dependent polarizability **α**(*t*) (finite systems) or susceptibility **χ**(*t*) (extended systems), e.g., in the case of the former
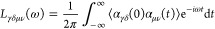
11Note that the elements of the polarizability (or susceptibility) tensor are selected by the polarization of the incoming and outgoing light as indicated in eq 10. Polarized Raman measurements can be directly related to eq 10 by combinations of the Raman line shape *L*(ω). One can also calculate an average spectrum for isotropic samples.^50^ The polarizability tensor (and equivalently the susceptibility tensor) can also be written as **α** = γ***I*** + **β** where γ = Tr(**α**)/3, and **β** is a traceless tensor to obtain the isotropic (polarized) and anisotropic (depolarized) spectrum. This leads to the decomposition
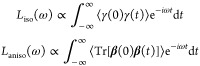
12

The electric susceptibility (Sect. 2.5) can be separated into an electronic and an ionic contribution
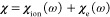
where the general frequency dependence of these terms is emphasized. For the prediction of Raman spectra, we only need to consider the electronic contribution **χ**_e_(ω). Furthermore, we limit ourselves to nonresonant Raman spectroscopy. This means that we require the electric susceptibility in the ion-clamped static limit, i.e., **χ**_e_(0), and do not have to consider the frequency dependence of **χ**_e_(ω), which arises from electronic transitions.

### 2.8 Workflow for Simulations of IR and Raman Spectra

By combining a NEP model for the PES with TNEP models for dipole, polarizability, or susceptibility, one obtains a simple yet general workflow for the computation of IR and Raman spectra (Figure 1). Starting from a NEP PES model, large-scale MD simulations are performed to sample the PES via the gpumd package, typically for a few hundred picoseconds. TNEP dipole, polarizability, or susceptibility models are then employed to predict **μ**(*t*), **α**(*t*), or **χ**(*t*) along the trajectory. Finally, IR or Raman spectra are obtained via Fourier transformation of the respective ACFs via eq 9 or 10.

## 3 Performance Evaluation

In this section, we evaluate the performance of the TNEP dipole, polarizability, and susceptibility models in comparison with models from the literature with respect to both regression accuracy and computational speed. The comparison includes the molecules H_2_O, (H_2_O)_2_, and H_5_O_2_^+^ (the Zundel cation), as well as a set of configurations representing liquid water. Structures with dipole, polarizability, and/or susceptibility data were retrieved from the repository maintained by the developers of the SA-GPR models^38,53^ (see Sect. S1 in the Supporting Information for details). The data set for each of these systems comprises 1000 configurations, half of which were use for training, while the other half were used for validation. The hyperparameters used in the training of the TNEP models are presented in Tables S1 and S2. In the case of the SA-GPR method, the results for liquid water were computed using a publicly available model,^54^ while the models for the molecules were trained by us (see Sect. S3 for details). In the case of the T-EANN method, we only use those data available in the literature.^39^

### 3.1 Dipole Moment

The TNEP dipole models can achieve very high precision when predicting **μ** for both molecules and liquid water with very low RMSEs (Table 1) and coefficients of determination (*R*^2^) very close to one (Figure S2).

**Table 1:** RMSEs (in e·bohr) and RRMSEs (unitless) for **μ** for the Validation Sets Using NEP Rank-1 Tensor Models^a^

System	RMSE	RRMSE
H_2_O	2 × 10^–4^	0.069%
(H_2_O)_2_	105 × 10^–4^	1.681%
H_5_O_2_^+^	14 × 10^–4^	0.371%
liquid water	17 × 10^–4^	0.852%

a For liquid water, the dipole moment is given per water molecule.

**Figure 2:**
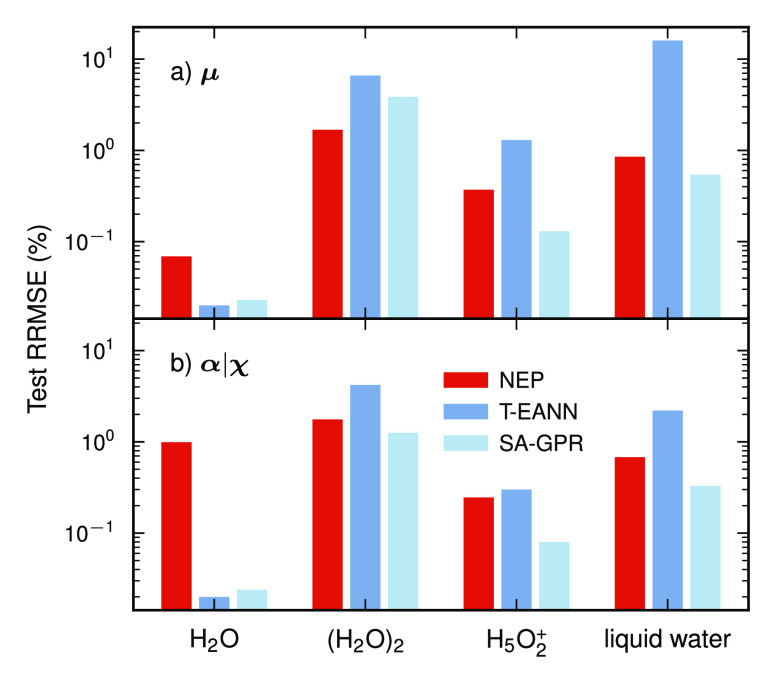
RRMSEs for the validation sets according to TNEP, T-EANN, and SA-GPR models for water systems for (a) **μ** as well as (b) **α** and **χ**/ρ. Validation RRMSEs for liquid water from T-EANN^39^ were reported for the averaged molecular polarizability obtained via the Clausius-Mossotti relation (S8). The validation RRMSEs for **χ**/ρ should be somewhat higher than that for the averaged molecular polarizability (also see Table S5).

As a further, more intuitive measure, one can also consider the root-mean-square-error relative to standard deviation (RRMSE),^39^ defined as the RMSE divided by the standard deviation of the reference data (Figure 2a). For the water monomer (H_2_O), all three methods yield extremely small RRMSEs below 0.1%. For the other three systems, including liquid water, the TNEP and SA-GPR models achieve comparable accuracy, while the T-EANN models perform systematically worse. This behavior is particularly pronounced for liquid water and might arise since the T-EANN model uses the positions relative to the center of mass as input, which are not well-defined in periodic systems.^55,56^

#### Neutral Molecules

The **μ** of neutral molecules such as H_2_O or (H_2_O)_2_ is uniquely defined. In the TNEP approach, **μ** is calculated by summing over atomic contributions which, in contrast to, e.g., the T-EANN approach, does not require choosing a reference point. Therefore, the TNEP dipole models are naturally suitable for neutral molecules.

In this context, we note that we also trained and validated a model for the QM7B data set containing thousands of neutral organic molecules,^57,58^ for which we make similar observations (Sect. S4). The TNEP model yields a very low RMSE for the validation set of 1.80 × 10^–3^ e·bohr atom^–1^ and a very high *R*^2^ score for the validation set of about 0.998.

#### Charged Molecules

The **μ** of charged molecules is nonunique and depends on the choice of the reference point.^4,59^ For charged molecules, one should therefore employ the relative permanent dipole **μ**_*r*_ defined with respect to the center of mass when training TNEP dipole models. The reference **μ** values in the H_5_O_2_^+^ data set^38,53^ have already been transformed to **μ**_*r*_. Therefore, the absolute dipole moment of H_5_O_2_^+^ including the movement of the center of mass should be **μ** = **μ**_*r*_ + *e*·***r***_COM_. The same procedure was applied to the PTAF^–^ molecule below (Sect. 4.3).

#### Periodic Systems

Traditional methods for calculating **μ** cannot be applied to periodic systems since the position operator is not uniquely defined.^56,60^ This issue is overcome via the modern theory of polarization,^38,60,61^ which provides a rigorous definition for the polarization of periodic systems and established a methodology for calculating **μ**. It was therefore used in the present work to obtain **μ** for periodic systems including water (Sect. S1) and α-Fe_2_O_3_ (Sect. S5). The TNEP model for α-Fe_2_O_3_ yields a very high *R*^2^ score for the validation set close to one.

### 3.2 Polarizability and Susceptibility

The RMSEs for the diagonal and off-diagonal elements of **α** of (H_2_O), (H_2_O)_2_, and H_5_O_2_^+^ are quite small (Table 2), indicating the high accuracy of the TNEP polarizability model. The coefficients of determination are larger than 0.98 mirroring this trend (Figure S7 and Figure S8). For liquid water, we consider **χ**/ρ, which has the unit of polarizability per atom. The RMSEs for **χ**/ρ are on the same order of magnitude as the RMSEs for **α** (Table 2).

**Table 2:** RMSEs (in bohr^3^) and RRMSEs (unitless) for **α** (Molecules) and **χ**/ρ (Liquid Water) for the Validation Sets Using TNEP Rank-2 Tensor Models^a^

	diagonal elements		off-diagonal elements	
System	RMSE	RRMSE	RMSE	RRMSE
H_2_O	85 × 10^–3^	5.89%	4 × 10^–3^	1.22%
(H_2_O)_2_	227 × 10^–3^	8.82%	137 × 10^–3^	12.59%
H_5_O_2_^+^	23 × 10^–3^	1.20%	17 × 10^–3^	1.06%
liquid water	54 × 10^–3^	16.28%	37 × 10^–3^	20.38%

a For liquid water, **χ**/ρ is given per water molecule.

The NEP models achieve an accuracy that is comparable to that of the T-EANN and SA-GPR models for the polarizability of (H_2_O)_2_ and H_5_O_2_^+^ as well as the susceptibility of liquid water (Figure 2b). While the performance for the water monomer H_2_O is worse, the TNEP model still yields a validation RRMSE of less than 1%.

As a further test, we constructed a TNEP polarizability model for the QM7B data set (Sect. S4). The RMSE values for the validation set are 4.64 × 10^–2^ bohr ^3^ atom^–1^ and 2.58 × 10^–2^ bohr ^3^ atom^–1^ for the diagonal and off-diagonal elements of **α**, respectively. For comparison, Wilkins et al.^62^ reported a higher RMSE value of 5.50 × 10^–2^ bohr ^3^ atom^–1^ over both the diagonal and off-diagonal elements of **α** using an SA-GPR model.

### 3.3 Computational Speed

It is now instructive to evaluate the computational performance of TNEP models in comparison with publicly available SA-GPR models.^53,54^ To this end, we consider liquid water systems with varying numbers of atoms. Starting from a cell containing 96 atoms, larger samples with up to 69984 atoms were created by replication.

The SA-GPR models can be run only serially on a central processing unit (CPU). In contrast, the TNEP model can be run on a CPU using NEP_CPU,^63^ e.g., via the interface provided by the calorine package,^64^ or on a graphics processing unit (GPU) by using the gpumd package. The SA-GPR and TNEP (CPU) models were tested on a server containing two Intel XEON Platinum 8275CL processors with a system memory of 256 GB, while the TNEP (GPU) models were tested on a heterogeneous server containing two Intel XEON Gold 6148 processors and an Nvidia GeForce RTX 4090 card with a graphics memory of 24 GB.

**Figure 3:**
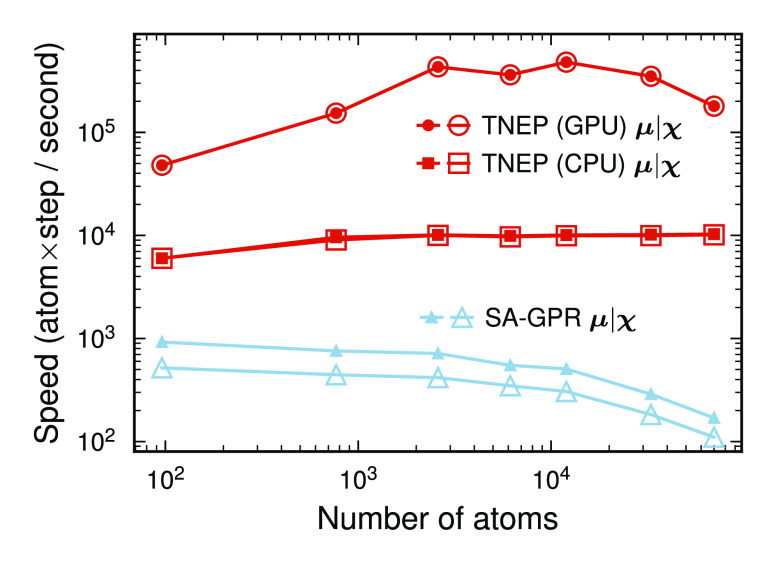
Comparison of computational speed of SA-GPR and TNEP models for the dipole (**μ**) and susceptibility (**χ**) of liquid water. Here, the SA-GPR results were obtained using the TENSOAP-FAST implementation.^54^

The comparisons show that for system sizes ≳1000 atoms the TNEP CPU models are at least 1 order of magnitude faster than the SA-GPR models on CPUs for both dipole and polarizability (Figure 3). On CPUs, the TNEP models exhibit nearly perfect weak scaling over the system sizes considered here. In contrast, the SA-GPR models show a notable decrease in speed as the system size increases. Running the TNEP models on GPUs enables an additional speedup of an order of magnitude or more. For very small systems, the GPU implementation is limited by IO. In addition, we note that gpumd allows one to evaluate TNEP models on-the-fly during MD simulations for prediction of tensorial properties with a small impact on simulation speed (Sect. S10).

## 4 Applications

Having established the accuracy and computational performance of the TNEP approach by comparison with reference data sets, we now demonstrate the application of NEP and TNEP models in combination for predicting the IR and Raman spectra of molecules, liquids, and solids. To this end, we employ the correlation function approach outlined above (Sect. 2.8 and Figure 1).

### 4.1 IR Spectrum of Water

First, we developed a NEP PES model for liquid water using energy, atomic forces, and virial data from density functional theory (DFT) calculations (Sect. S2).

Next, a system of 216 water molecules was equilibrated in the NPT ensemble for 100 ps using the trained PES model at 298 K and 1 bar, followed by a further equilibration run in the NVT ensemble for another 100 ps. Three production runs were carried out in the NVE ensemble for a duration of 200 ps. A time step of 0.5 fs was used throughout. We note that quantum effects can be actually rather pronounced in water as has been shown by path integral MD simulations in, e.g., refs.^65−67^ Here, however, we decided to carry out classical MD simulations in order to enable a one-to-one comparison with the results of earlier studies.

The time dependence of the dipole (**μ**(*t*)) was computed for the production trajectories with a spacing of 1 fs using the TNEP dipole model for liquid water described above (Sect. 3.1). The IR spectrum was then obtained by Fourier transform of the dipole moment ACF via eq 9. The final IR spectrum was obtained by averaging the IR spectra from the production runs.

For comparison, we also ran a 200 ps MD simulation with the TIP3P force field^68^ via the CP2K software package,^69^ where the TIP3P force field uses charges of −0.834 e and +0.417 e for oxygen and hydrogen, respectively.

**Figure 4:**
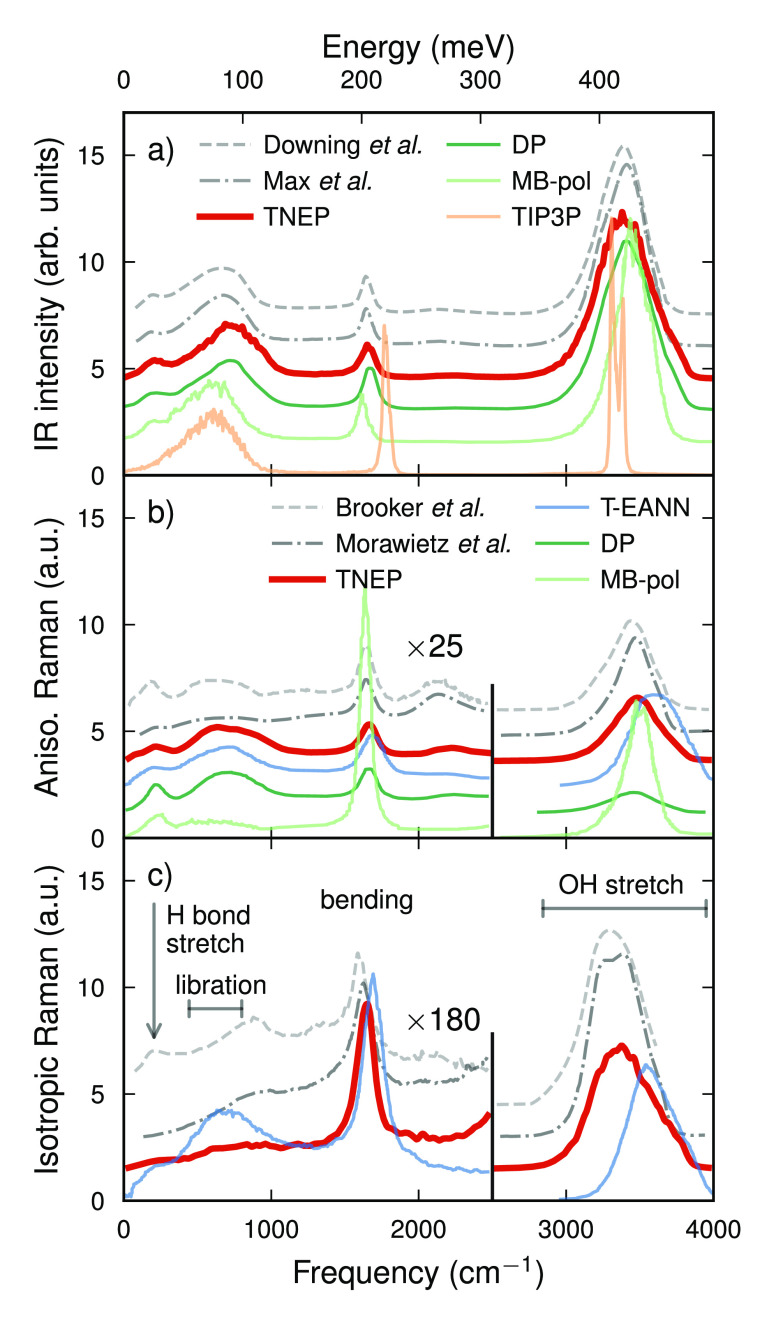
Comparison of (a) infrared as well as (b) anisotropic (depolarized) and (c) isotropic (polarized) Raman spectra of water at ambient conditions from simulations and experiment. Experimental data from Downing et al.,^70^ Max et al.,^71^ Brooker et al.,^76^ and Morawietz et al.^77^ Simulated spectra from T-EANN,^39^ MB-pol,^37^ and DP^10,44^ models were adapted from the literature. In (a) and (b), the spectra were normalized by the integral between 80 and 2500 cm^–1^, while in (c), they were normalized by the integral between 1000 and 2500 cm^–1^.

The NEP-TNEP method yields an IR spectrum that is in very good agreement with experimental data^70,71^ over the entire frequency range from 0 to 4000 cm^–1^ (Figure 4a). This includes the hydrogen-bond stretching band^10^ between 160 and 250 cm^–1^, the libration band^10^ from 400 to 800 cm^–1^ associated with hindered molecule rotations,^37^ and the bending modes^37,72^ at about 1650 cm^–1^ as well as the OH stretching band^37,72^ from 2800 to 4000 cm^–1^. The NEP and TNEP models for PES and **μ** in conjunction with the underlying exchange-correlation functional thus succeed in capturing the entire range stretching from soft intermolecular to stiff intramolecular modes. This performance is also observed for the DP model (Figure 4a).

By comparison, classical models produce rather large errors for the location of several features in the IR spectrum of water. MD simulations with classical force fields^68,73^ such as TIP3P (Figure 4a) and SPC/E tend to predict a blue-shifting of the bending modes by roughly 100 to 200 cm^–1^. A similar tendency was also observed for the POLI2VS model.^36^ The results from the MB-pol model on the other hand exhibit a blue-shift of the OH stretching band by about 50 cm^–1^.^37^

The width of the OH stretching band has been proven to be quite difficult to predict due to the anharmonicity of the OH stretch mode.^37^ The NEP-TNEP approach yields a value of 380 cm^–1^ for the full width at half-maximum of this band, which is in good agreement with experimental estimates of about 350 cm^–1^ from Downing et al.^70^ Both NEP-TNEP and DP predictions exhibit a slight high-frequency tail for this band, which is not visible in the experimental spectra. This small difference could originate from the strongly constrained and appropriately normed (SCAN) functional^74^ that was used for generating the PES training data^10,75^ and/or the absence of quantum effects in the (classical) MD simulations.^10,37^

### 4.2 Raman Spectra of Water

To obtain the Raman spectra of liquid water, we sampled the time dependence of **χ**(*t*) using the TNEP susceptibility model and subsequently computed the ACFs for the same trajectories used for the prediction of the IR spectra. The full spectrum given by eq 10 and averaged over the available trajectories was then split into isotropic (polarized) and anisotropic (depolarized) contributions via eq 12.

The **anisotropic spectrum** predicted by the NEP-TNEP approach is overall in very good agreement with experimental data (Figure 4b).^76,77^ The locations of the peaks and relative intensities of the stretching, bending, and librational modes in the simulated anisotropic Raman spectra are all well produced. It is noteworthy that in the low frequency region below approximately 1000 cm^–1^, the variation between the experimental spectra is larger than the variation between the ML models and the experimental data. This could be related to difficulties associated with processing the experimental raw data in this frequency region.

The T-EANN and DP models yield results similar to those of the NEP-TNEP approach in the region up to about 1900 cm^–1^. On the other hand, all ML models underestimate the intensity of the association band between 1900 and 2500 cm^–1^, which is arising from the combination of librational and bending modes.^37,77^ Here, the NEP-TNEP prediction is actually still the one that comes closest to the experimental spectra.

The broad high-frequency peak above 3000 cm^–1^, which is associated with the OH stretch mode, is notably blue-shifted and broadened for the T-EANN model, while the DP model strongly underestimates the intensity of this peak. In contrast, the NEP-TNEP combination predicts this feature in good agreement with the experimental data.

Finally, the parametric MB-pol model yields the worst agreement with experiment, for example, strongly overestimating the intensity of the bending band while underestimating the libration band.

With regard to the **isotropic Raman spectrum** (Figure 4c), one should first note the variation among the experimental data. In particular, in the region below 1000 cm^–1^, the resulting uncertainty is comparable or even larger than the deviation between the NEP-TNEP prediction and the experimental data, while the position of the libration band predicted by T-EANN appears red-shifted. With regard to the higher frequency region, both NEP-TNEP and T-EANN reproduce the bending band well. In the case of NEP-TNEP, this also applies for the OH stretch band, whereas in the case of T-EANN, a blue-shift can be observed similar to that of the anisotropic spectrum (Figure 4b).

### 4.3 IR Spectrum of PTAF^–^

**Figure 5:**
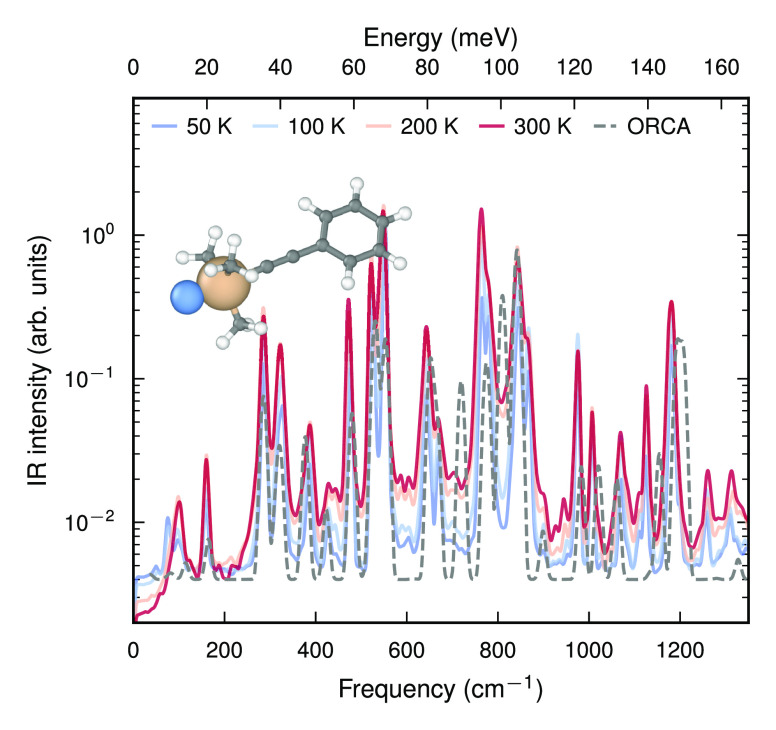
IR spectra for the metastable PTAF^–^ complex (see the inset) at various temperatures. The gray dashed line represents the broadened integrated absorption coefficients of the harmonic spectrum obtained directly from DFT calculations. The overall agreement is good considering the lack of anharmonic corrections (intensity and vibrational frequencies) and temperature sensitivity of the spectrum obtained from DFT calculations.

The NEP-TNEP method for predicting IR spectra can be easily adopted for other molecular systems as long as the underlying observables to be learned are available. Naturally, this includes the molecular configurations along a chemical reaction such that experimentally observable spectral changes can be connected to metastable complexes. One such complex is PTAF^–^ (see inset in Figure 5), the intermediate reaction minimum in the deprotection reaction 1-phenyl-2-trimethylsilylacetylene (PTA) with tetra-n-butylammonium fluoride.^78−81^

To train NEP and TNEP models, we obtained PES and **μ** data for a set of 20170 structures via DFT calculations using the ORCA code,^82^ the PBE functional,^83^ and a def2-TZVP basis set^84^ while enforcing tight convergence of the self-consistent field cycles. Subsequently, MD simulations at various temperatures were performed in the NVE ensemble using a time step of 0.1 fs for 1 ns, during which **μ**(*t*) was recorded with a time resolution of 0.5 fs.

The IR spectra obtained via the analysis of the ACF of **μ** show a pronounced temperature dependence in particular of the line widths (Figure 5). The molecule supports several soft modes with frequencies in the region below 250 cm^–1^, which are associated with the bending of and rotation about the ethynyl linker. These modes in particular lead to strong mode coupling (i.e., anharmonicity), which underlies the changes in line width and the redistribution of the dipole strength across the spectrum. Here, the computational efficiency of the NEP-TNEP implementation in gpumd was crucial to resolve these features, as it enabled sampling on the nanosecond time scale, which would be prohibitive for DFT-MD simulations and computationally very expensive for a CPU implementation.

### 4.4 Raman Spectra of BaZrO_3_

BaZrO_3_ is a perovskite that is being investigated, e.g., as a proton conductor for applications in fuel cells. It has also been the subject of various fundamental studies, as it is a prototypical antiferroelectric perovskite.^85−87^ It features soft and strongly temperature-dependent phonon modes,^88,89^ which have been carefully analyzed with Raman spectroscopy,^90^ rendering BaZrO_3_ an ideal application for the present approach.

For benchmarking, we constructed models for **χ** using both the TNEP and SA-GPR approaches. The reference data set comprised cubic and tetragonal supercells with up to 40 atoms. The training structures were taken from MD simulations at different temperatures and pressures, generated using a NEP PES model constructed in an earlier study.^89^ In total, the reference data set contained 940 structures. 140 structures were randomly placed in a holdout set for validation, while training sets were compiled by the shuffle-split method (random selection with replacement) with 200 to 800 structures and five data sets per training set size.

A comparison of models generated using different choices for the size of the neural network as well as the descriptor demonstrates that viable models can be obtained for a wide range of parameters and that even small models with as few as 1500 or so parameters can yield very good results (Figure S12). Yet fine-tuning of these parameters as well as the regularization parameters (Figure S13) allows one to maximize model performance.

**Figure 6:**
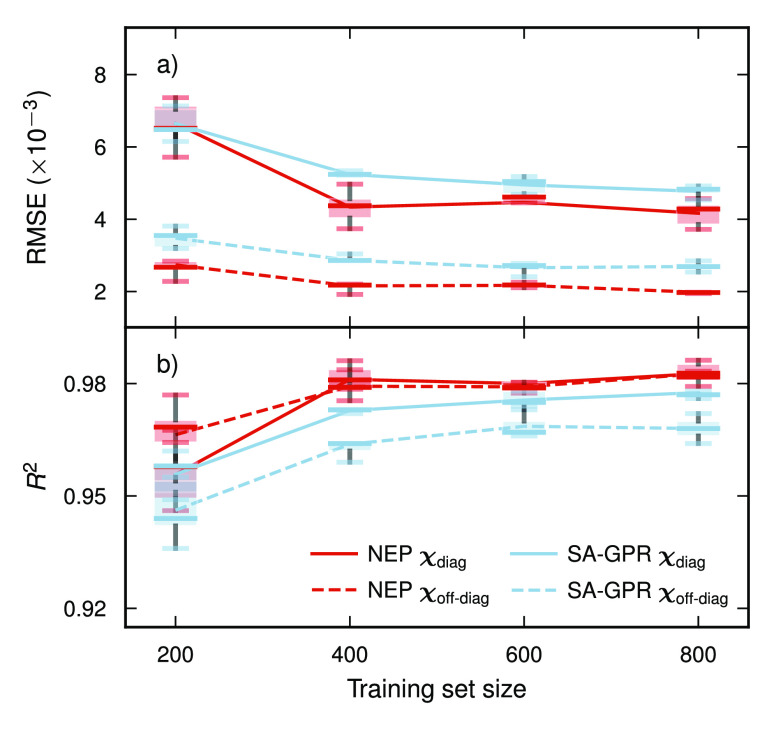
Variation of (a) RMSEs and (b) *R*^2^ scores with training set size for TNEP and SA-GPR models based on five training sets per size generated by shuffle-split. In the case of TNEP, we used *N*_neu_ = 20, *n*_max_^R^ = *n*_max_^A^ = 4, and λ_1_ = λ_2_ = 2 × 10^–3^ (compare Figures S12 and S13).

The convergence of RMSEs and *R*^2^ scores with training set size is similar for TNEP and SA-GPR with slightly better performance for TNEP (Figure 6). In both cases, training sets of about 400 structures already yield very good models, demonstrating the data efficiency of these approaches. This behavior has also been observed in the construction of models for amino acids.^91^

**Figure 7:**
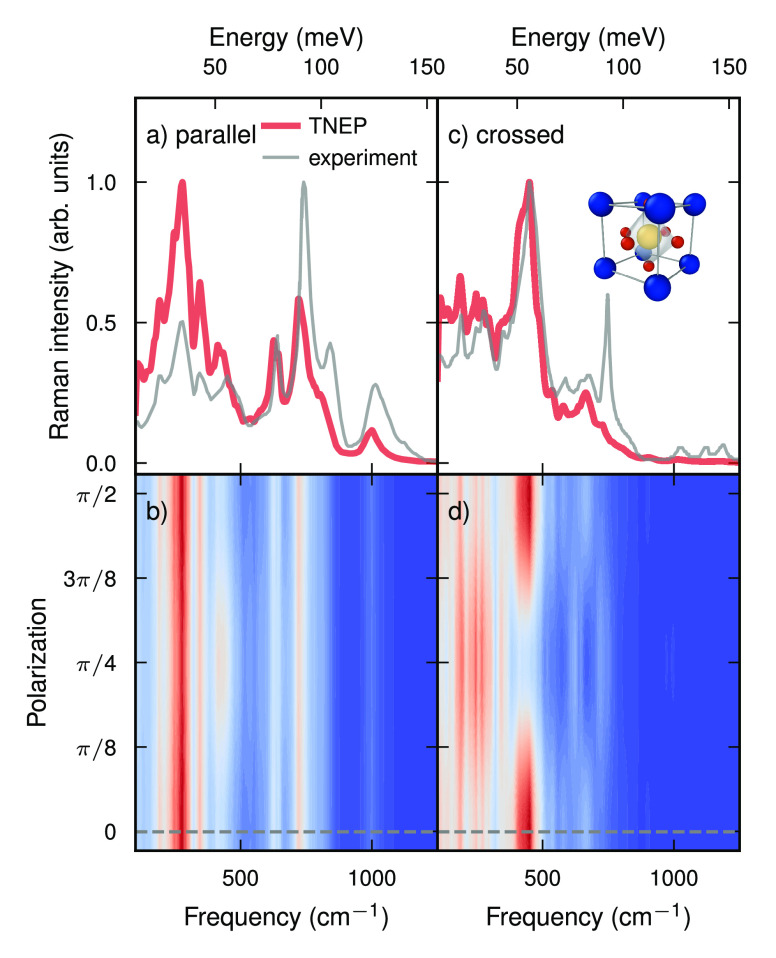
Raman spectra of BaZrO_3_ for (a,b) parallel and (c,d) crossed polarization from simulations using a combination of NEP and TNEP models (red lines) as well as experiment (gray lines).^90^ The spectra shown in (a,c) have been predicted for the nominal alignments used in the experimental measurements. The corresponding polarizations are indicated by the dashed horizontal lines in (b,d).

Next MD simulations were carried out using 12 × 12 × 12 supercells (8640 atoms) and a time step of 1 fs using the NEP model for the PES. Following equilibration at 300 K and 0 GPa in the NPT ensemble, the time-dependent susceptibility **χ**(*t*) was recorded for 500 ps using a time resolution of 5 fs. For production, we used a TNEP model for **χ** trained against the full data set, but we found that models based on at least approximately 400 structures yield results that are practically indistinguishable within the statistical uncertainty. The Raman line shape was subsequently obtained via the ACF of **χ** according to eq 11. We then computed the Raman spectra for parallel (Figure 7a,b) and crossed polarization (Figure 7c,d), which in Porto notation correspond to *Z*(*XX*) *Z̅* and *Z*(*XY*)*Z̅*, respectively, where *X* and *Y* are arbitrary crystal axes. The final spectra were obtained by averaging over 20 independent MD trajectories.

The results are overall in very good agreement with experiment, especially considering the very strong anharmonicity of this material and the strong temperature dependence of the vibrational spectrum.^88^ The main difference with respect to the position of the peaks is a slight red-shift in the predicted spectra in the region above 600 cm^–1^. This overly soft response can be attributed to the underlying exchange-correlation functional (vdW-DF-cx, refs.^92,93^), which the NEP model truthfully reproduces. One can also observe an inversion in the intensity of the low- and high energy features. This effect is almost certainly due to the classical sampling used here. It is rather common to correct for quantum effects in IR and *first order* Raman spectra by including a factor similar to the prefactor in eq 8. In the case of BaZrO_3_ the room-temperature Raman spectrum arises, however, due to *second-order* scattering, i.e., due to combinations of modes. In that case, the application of the commonly used correction factor is no longer valid. Here, we omit such corrections entirely.

The Raman spectra depend on the crystal orientation with respect to the excitation laser. The present approach allows one to readily map out this dependence via eqs 10 and 11 (Figure 7b,d). While we are unaware of experimental measurements of the polarization dependence for BaZrO_3_, we note that such experiments have been carried out for, e.g., NaCl.^94^ As demonstrated in the previous study, such measurements can provide valuable additional information.

## 5 Conclusions

In this contribution, we introduced an extension of the NEP approach to tensors, resulting in the TNEP scheme. This was achieved by constructing expressions for rank-1 and rank-2 tensors based on the expression for the virial, which is a rank-2 tensor that arises naturally from derivatives of the energy (a rank-0 tensor) with respect to the atomic distances. This approach, which can be extended to tensors of higher rank, thus allows one to easily construct models that are equivariant.

We demonstrated the accuracy of this approach and its computational efficiency by constructing models for the dipole moment **μ**, the molecular polarizability **α**, and the electric susceptibility **χ** for several molecules, a liquid, and two crystalline materials. In particular, the computational speed of the current method and its implementation in the gpumd package provide a significant advantage in terms of both the time scales and system sizes that can be sampled.

Finally, we applied the approach to predict IR and Raman spectra of liquid water, the molecule PTAF^–^, and the perovskite BaZrO_3_ in very good agreement with available experimental data, illustrating the range of systems that can be readily addressed by using the TNEP methodology introduced here.

## Data Availability

The source code and documentation for gpumd are available at https://github.com/brucefan1983/GPUMD and https://gpumd.org, respectively. The source code and documentation for calorine are available at https://gitlab.com/materials-modeling/calorine and https://calorine.materialsmodeling.org, respectively. NEP models and data are available via Zenodo via 10.5281/zenodo.10257363 (water and BaZrO_3_), 10.5281/zenodo.10255268 (PTAF^–^), and 10.5281/zenodo.8337182 (BaZrO_3_).

## Supporting Information Available

Training process of SA-GPR models for water systems; calculations of dipole moment for liquid water and α-Fe_2_O_3_; notes on the Clausius-Mossotti relation and the units of polarizability and electric susceptibility; parity plots of the TNEP predicted dipole moment, diagonal and off-diagonal elements of the polarizability tensor versus the *ab initio* references for water systems; demonstration of rotational invariance; parameters used in the training of NEP and TNEP models; dependence of TNEP model performance for BaZrO_3_ on model and training hyperparameters; timing of on-the-fly model evaluation during MD simulations (PDF)
