# Supplementary material for: Tensorial Properties via the Neuroevolution Potential Framework: Fast Simulation of Infrared and Raman Spectra
Source: J Chem Theory Comput. 2024 Apr 4;20(8):3273–84. doi: 10.1021/acs.jctc.3c01343 (PMC11044275; doi:10.1021/acs.jctc.3c01343)
Supplement: Supplementary file 1 — ct3c01343_si_001.pdf [file ct3c01343_si_001.pdf]

Supporting Information:

Tensorial properties via the neuroevolution potential framework: Fast simulation of infrared and Raman spectra

Nan Xu[1,2], Petter Rosander[3], Christian Schäfer[3], Eric Lindgren[3], Nicklas Österbacka[3], Mandi Fang[1,2], Wei Chen[4], Yi He[1,2,5,*], Zheyong Fan[6,†], and Paul Erhart[3,‡]

[1] *Institute of Zhejiang University-Quzhou, Quzhou 324000, P. R. China*
[2] *College of Chemical and Biological Engineering, Zhejiang University, Hangzhou 310058, P. R. China*
[3] *Department of Physics, Chalmers University of Technology, SE-41296, Gothenburg, Sweden*
[4] *State Key Laboratory of Multiphase Complex Systems, Institute of Process Engineering, Chinese Academy of Sciences, Beijing, P. R. China*
[5] *Department of Chemical Engineering, University of Washington, Seattle, WA 98195, USA*
[6] *College of Physical Science and Technology, Bohai University, Jinzhou 121013, P. R. China*
[*] *yihezj@zju.edu.cn*
[†] *brucenju@gmail.com*
[‡] *erhart@chalmers.se*

March 19, 2024

Contents

S1 Dipole, polarizability, and susceptibility data for water systems 2

S2 Training of NEP PES model for water 2

S3 Training of SA-GPR models for water molecules 2

S4 Training of TNEP models for QM7b data set 3

S5 Training of TNEP dipole model for α-Fe_2O_3 3

S6 Training of TNEP susceptibility model for $BaZrO_3$ 3

S7 Note on units of polarizability and susceptibility 4

S8 Note on the Clausius-Mossotti relation 4

S9 Rotational equivariance of TNEP models 4

S10 On-the-fly prediction of dipoles and polarizability during molecular dynamics (MD) simulations 5

S1 Dipole, polarizability, and susceptibility data for water systems

Data for the dipole moment ($\boldsymbol{\mu}$), the polarizability ($\boldsymbol{\alpha}$), and the electronic part of the electric susceptibility ($\boldsymbol{\chi}_e$) for molecular water species as well as liquid water were retrieved from a public repository.[1,2] The $\boldsymbol{\mu}$ and $\boldsymbol{\alpha}$ data for the molecules H_2O, $(H_2O)_2$, and ${H_5O_2}^+$ provided in the repository were calculated at the coupled cluster singles and doubles (CCSD) level of theory[3,4] with the d-aug-cc-pVTZ[5,6] basis set. The data for liquid water in the repository were generated using the Perdew–Burke–Ernzerhof (PBE)[7] functional and ultra-soft pseudo-potentials (USPPs).[8] The repository contains data for the relative dielectric permittivity $\boldsymbol{\varepsilon}_r$. Here, the latter was converted to the electric susceptibility $\boldsymbol{\chi}_e = \boldsymbol{\varepsilon}_r - \mathbf{1}$ for training.

We demonstrate here two approaches to calculate dipole moment for periodic systems (liquid water) via the modern theory of polarization[2,9,10]. A total of 50 structures was randomly selected from the liquid water data set. In the first approach, the total $\boldsymbol{\mu}$ of each structure was calculated using the Berry phase formulation[9]. The calculations were performed using the CP2K software package[11] with density functional theory (DFT) implemented using the gaussians and plane waves (GPW) method. The TZV2PX-MOLOPT-GTH basis set and GTH-PBE pseudopotentials were used.[7,12] In addition, the DFT-D3 correction[13] was employed to capture dispersive van-der-Waals interactions. As shown in Fig. S4a, most of the calculated $\boldsymbol{\mu}$ are consistent with the reference $\boldsymbol{\mu}$, while some of them have significant offsets. These $\boldsymbol{\mu}$ data points were shifted by $N \cdot L$, where N is an integer and L is the lattice parameter, thereby account for the phase shift that arises in the modern theory of polarization[9]. As shown in Fig. S4b, the shifted $\boldsymbol{\mu}$ data points are consistent with the reference $\boldsymbol{\mu}$ data. In the second approach, the total $\boldsymbol{\mu}$ of each structure was calculated using maximally localized Wannier functions (MLWFs)[14]

$$\boldsymbol{\mu} = 6\mathrm{e}\sum_i r_i^{\mathrm{O}} + \mathrm{e}\sum_j r_j^{\mathrm{H}} - 2\mathrm{e}\sum_k r_k^{\mathrm{MLWF}},$$

where r_i^{O} and r_j^{H} are the coordinates of oxygen and hydrogen atoms and r_k^{MLWF} is the coordinate of the Wannier centers belonging to a water molecule.[15] In this case, a proper coordinate transformation of the oxygen and hydrogen atoms as well as the Wannier centers is required before calculating the total dipole moment.(Fig. S5) As shown in Fig. S4c, the $\boldsymbol{\mu}$ data points calculated from MLWFs are consistent with the reference $\boldsymbol{\mu}$ data.

S2 Training of NEP PES model for water

We used the potential energy surface (PES) data set for liquid water from Ref. 16,17 to train a neuroevolution potential (NEP) PES model to be used for MD simulations. The data set contains 1888 structures in total, for which energy, forces, and virials have been computed using DFT calculations. The strongly constrained and appropriately normed (SCAN)[18] functional and the projector augmented wave (PAW) method[19] (with hard setups) were used. All data were randomly divided into training and validation data sets with a ratio of 4:1. The hyperparameters used in the training of the NEP model are tabulated in Table S3. The root-mean-square errors (RMSEs) for energy, forces, and virials converged after 3×10^5 generations of training, and the predicted energies, forces, and virials closely match the DFT reference data, as shown in Fig. S9. We also performed an NPT (isothermal-isobaric) MD simulation of liquid water (64 molecules) using the NEP PES model at 330 K and 1 bar. The radial distribution functions (RDFs) for O–O and O–H pairs extracted from these simulations agree well with *ab initio* molecular dynamics (AIMD) simulation in the literature[20] (Fig. S10).

S3 Training of SA-GPR models for water molecules

Since we did not find publicly accessible models for H_2O, $(H_2O)_2$ and ${H_5O_2}^+$, we trained new symmetry-adapted Gaussian process regression (SA-GPR) models for $\boldsymbol{\mu}$ and $\boldsymbol{\alpha}$ using the same data sets as those used for training the tensorial neuroevolution potential (TNEP) models (Sect. S1). The default hyperparameters were used in the training.

S4 Training of TNEP models for QM7b data set

We also consider the QM7b data set[21] that comprises 7211 small organic molecules with up to six elements (H, C, N, O, S, Cl). The reference $\boldsymbol{\mu}$ and $\boldsymbol{\alpha}$ data[22] were calculated at the CCSD level of theory using the d-aug-cc-pVDZ basis set[5,6]. 70% of the data were use for training, while the remaining data were used for validation. The parameters in the training of the TNEP dipole and polarizability models are shown in Table S1 and Table S2. Both the dipole and polarizability models achieve very high precision for the QM7B data set (Fig. S3).

S5 Training of TNEP dipole model for α-Fe_2O_3

We also consider yet another crystalline system in α-Fe_2O_3. The primitive structure of α-Fe_2O_3 reported by Mendili *et al.*[23] was used here, which is defined by three vectors: $\boldsymbol{a} = (5.03, 0.00, 0.00)$, $\boldsymbol{b} = (-2.515, 4.356, 0.00)$, and $\boldsymbol{c} = (0.00, 0.00, 13.75)$, measured in units of Å. A $2 \times 2 \times 1$ supercell was created and used as the initial structure for AIMD simulations. The simulation system contained 120 atoms. The AIMD simulations were performed using a time step of 0.5 fs and the CP2K software package[11]. The forces on the atoms were evaluated using the DZVP-GTH-PADE basis set and GTH-PADE pseudopotentials[24,25]. To maintain the temperature at 300 K, a Nosé-Hoover thermostat was employed[26]. During the simulation, a magnetic moment of $5\,\mu_B$ was assigned to each iron atom, where μ_B represents the Bohr magneton. The simulation was run for a total of 2000 time steps, and snapshots were saved with an interval of 1 fs. In total, 1000 structures were sampled and used for post-processing. The $\boldsymbol{\mu}$ data of the 1000 structures were calculated using the Berry phase formulation,[9] where the origin of the coordinate system was used as the reference point. The calculated values vary continuously with the simulation time, indicating that all configurations are on the same branches of the Berry phase and no shifts are required for calculating $\boldsymbol{\mu}$ (Fig. S6a). The 1000 data points were randomly divided into a training data set and a validation data set with a ratio of 7:3. The hyperparameters used for training the TNEP dipole model are tabulated in Table S1. The $\boldsymbol{\mu}$ values predicted by the TNEP model are consistent with the DFT reference values (Fig. S6b). The coefficient of determination (R^2) is close to one.

S6 Training of TNEP susceptibility model for $BaZrO_3$

The training data points were taken from MD snapshots at various temperatures and pressures obtained using a previously published NEP model for the $BaZrO_3$ PES.[27] Both the cubic and tetragonal phases were included, with supercell sizes varying from the primitive cell to $4 \times 2 \times 1$ repetitions. The final dataset consisted of 940 structures, for which the relative permittivity $\boldsymbol{\varepsilon}_r$ was obtained from DFT calculations using the PAW formalism[19,28] as implemented in the Vienna Ab-initio Simulation Package[29,30]. The van-der-Waals density functional with consistent exchange (vdW-DF-cx) was used to describe the exchange-correlation energy contribution[31,32]. A plane-wave energy cutoff of of 510 eV was used along with Gaussian smearing with a width of 0.01 eV. Projection operators were evaluated in reciprocal space and an additional support grid was used for evaluation of augmentation charges for increased accuracy.

The hyperparameters used for training the TNEP models for $BaZrO_3$ are tabulated in Table S2. The relative permittivity was converted to electric susceptibility via the relation $\boldsymbol{\chi}_e = \boldsymbol{\varepsilon}_r - \mathbf{1}$ for training. Five-fold leave-one-out cross validation was carried out, where the dataset was split into five equal parts. Five separate models were then trained, each using one of these splits as a validation set. These models were trained until their validation set RMSE stopped improving, which occurred after 6×10^5 generations. Their mean coefficient of determination (R^2) was 0.950(2) for the diagonal and 0.984(2) for the off-diagonal elements. These quantities serve as accuracy estimates for the final model, which was trained on the entire dataset. Training of this model was considered converged after 6×10^5 generations based on the cross validation result, after which the TNEP model predicted values for the susceptibility consistent with DFT reference values (Fig. S11).

S7 Note on units of polarizability and susceptibility

For a single molecule, the *molecular polarizability* $\boldsymbol{\alpha}$ connects the induced dipole moment $\boldsymbol{\mu}_{\text{ind}}$ (charge times distance per molecule) to the electric field $\boldsymbol{E}$ (potential per distance),

$$\boldsymbol{\mu}_{\text{ind}} = \boldsymbol{\alpha}\boldsymbol{E}. \tag{S1}$$

For a bulk material, the *electric susceptibility* $\boldsymbol{\chi}$ connects the polarization (or dipole density) $\boldsymbol{P}$ (charge times distance per volume) to the electric field $\boldsymbol{E}$ (potential per distance),

$$\boldsymbol{P} = \epsilon_0\boldsymbol{\chi}\boldsymbol{E}. \tag{S2}$$

It is now instructive to consider the different units involved in the expressions above. Here, we explicitly included the unit mol, which is strictly part of the SI system but often left out. This is done in order to emphasize the transition from molecular quantities ($\boldsymbol{\alpha}$) to bulk quantities ($\epsilon_0\boldsymbol{\chi}$). This occurs, for example, in the Clausius-Mossotti relation, where $\boldsymbol{\alpha}$ is related to $\epsilon_0\boldsymbol{\chi}$ by scaling with the number volume density ρ. Here, we see that $\boldsymbol{\alpha}$ has units of $\mathrm{F\,m^2/mol}$ whereas $\epsilon_0\boldsymbol{\chi}$ has units of F/m and thus units of $\boldsymbol{\alpha}$ per volume. The following table summarizes the SI units of the quantities as defined above.

Quantity	SI unit
$\boldsymbol{\mu}_{\text{ind}}$	$\mathrm{C\,m/mol}$
$\boldsymbol{E}$	$\mathrm{V/m}$
$\boldsymbol{\alpha}$	$\mathrm{C\,m^2/(V\,mol) = F\,m^2/mol}$
$\boldsymbol{P}$	$\mathrm{C/m^2}$
$\boldsymbol{\epsilon}_0$	$\mathrm{F/m}$
$\boldsymbol{\chi}$	1
ρ	$\mathrm{mol/m^3}$

We note that there are various ways in which the relations Eqs. (S1) and (S2) are written in the literature. One can for example subsume ϵ_0 into $\boldsymbol{\chi}$, which emphasizes the symmetry with $\boldsymbol{\alpha}$. One can also choose to express $\boldsymbol{\alpha}$ in units of ϵ_0, i.e., $\boldsymbol{\alpha} = \epsilon_0\boldsymbol{\alpha}'$, in which case $\boldsymbol{\alpha}'$ has units of volume.

S8 Note on the Clausius-Mossotti relation

For non-polar liquids or gases, the Clausius-Mossotti relation[33] can be used to approximate the relation between the average molecular polarizability $\alpha = \mathrm{Tr}(\boldsymbol{\alpha})/3$ and the average electric susceptibility $\chi = \mathrm{Tr}(\boldsymbol{\chi})/3$. It is based on a mean-field approximation to account for local field effect and is given by

$$\frac{\chi_e}{\chi_e + 3} = \frac{\rho\alpha}{3\epsilon_0},$$

where ρ is the number volume density. The quantities are expressed in SI units. The Clausius-Mossotti can also be written in terms of the relative permittivity ε_r, which is related to the electric susceptibility via $\varepsilon_r = 1 + \chi$.

Note that all quantities here are isotropic and therefore represented by scalars. Here, α is the molecular polarizability, ρ is the number volume density, ϵ_0 is the permittivity of vacuum, and ε_r is the relative permittivity.

S9 Rotational equivariance of TNEP models

Here, we explicitly demonstrate that the TNEP dipole and polarizability model are invariant to rotations by taking the water monomer (H_2O) as an example. Dipole moments ($\boldsymbol{\mu}$) and polarizabilities ($\boldsymbol{\alpha}$) were predicted for the validation set using the TNEP models described in the manuscript. Then, structures were subjected to rotations ($\mathbf{R}$) at random angles. Dipole moments ($\boldsymbol{\mu}'$) and polarizabilities

($\boldsymbol{\alpha}'$) of the rotated structures were also predicted using the TNEP models. According to the rotational symmetries of rank-1 and rank-2 tensors, the rotational transformed dipole moments ($\boldsymbol{\mu}''$) and polarizabilities ($\boldsymbol{\alpha}''$) should be $(\mathbf{R}\boldsymbol{\mu}^{\mathrm{T}})^{\mathrm{T}}$ and $\mathbf{R}\boldsymbol{\alpha}\mathbf{R}^{-1}$, respectively. The parity plots of $\boldsymbol{\mu}''$ and $\boldsymbol{\alpha}''$ versus $\boldsymbol{\mu}'$ and $\boldsymbol{\alpha}'$ for the validation set of H_2O clearly demonstrate the expected rotational invariance (Fig. S14).

S10 On-the-fly prediction of dipoles and polarizability during MD simulations

Predicting dipoles or polarizabilities can be efficiently performed on-the-fly during MD runs in GPUMD. Fig. S15 shows how the performance of MD simulations scales with system size when tensorial properties are predicted at every tenth timestep, compared to a baseline without any tensorial predictions. Note that the overhead of evaluating tensorial properties on-the-fly during MD of course depends on how often they are to be evaluated, i.e., the number of timesteps between predictions.

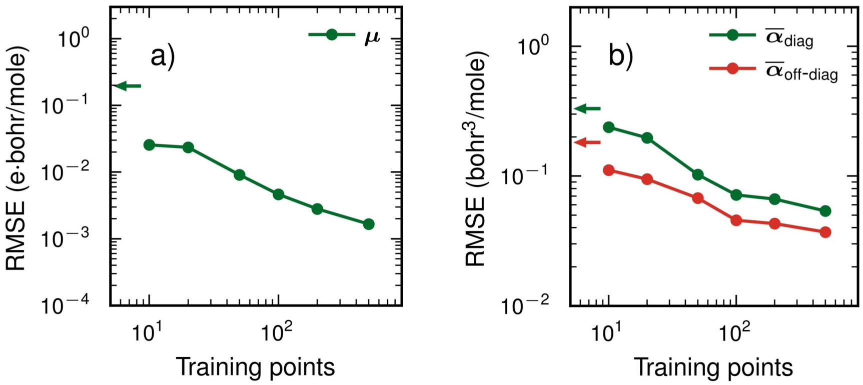

Figure S1: The learning curves for (a) dipole moment and (b) effective polarizability of liquid water. The effective polarizability is given by $\bar{\boldsymbol{\alpha}} = \boldsymbol{\chi}/\rho$, where ρ in the number volume density. For all cases, the validation data set consists of 500 independent configurations. Arrows indicate the intrinsic standard deviation of the validation data set.

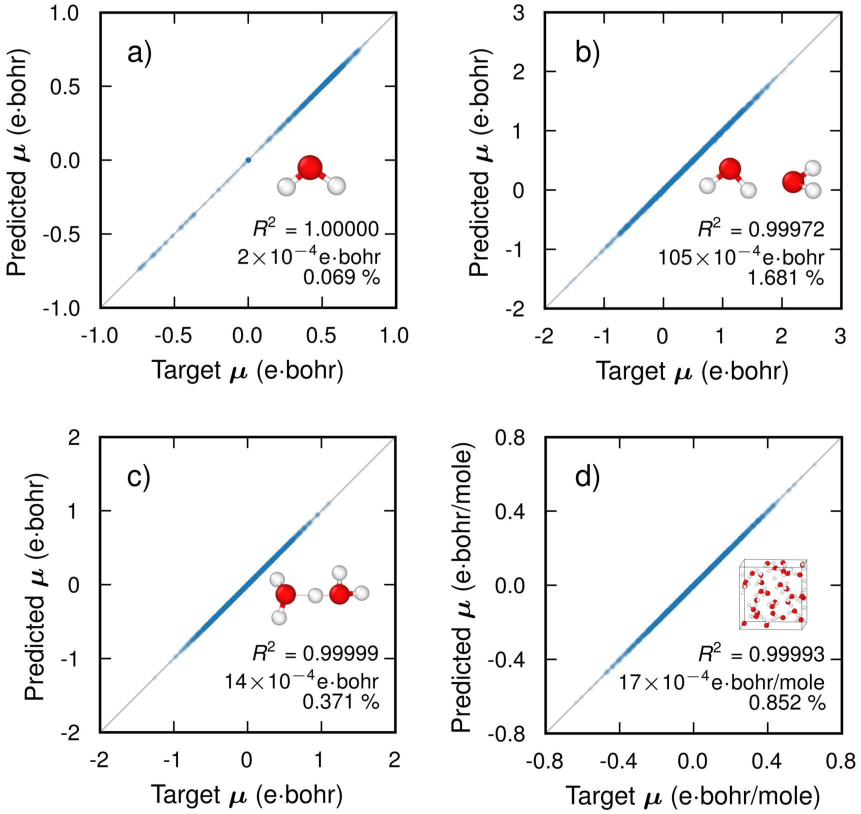

Figure S2: TNEP predicted dipole moment compared to *ab initio* data for the validation sets of (a) H_2O, (b) $(H_2O)_2$, (c) $H_5O_2{}^+$, and (d) liquid water. The coefficients of determination (R^2), RMSEs and RRMSEs are indicated in each subpanel. For liquid water, the dipole moment is given per water molecule.

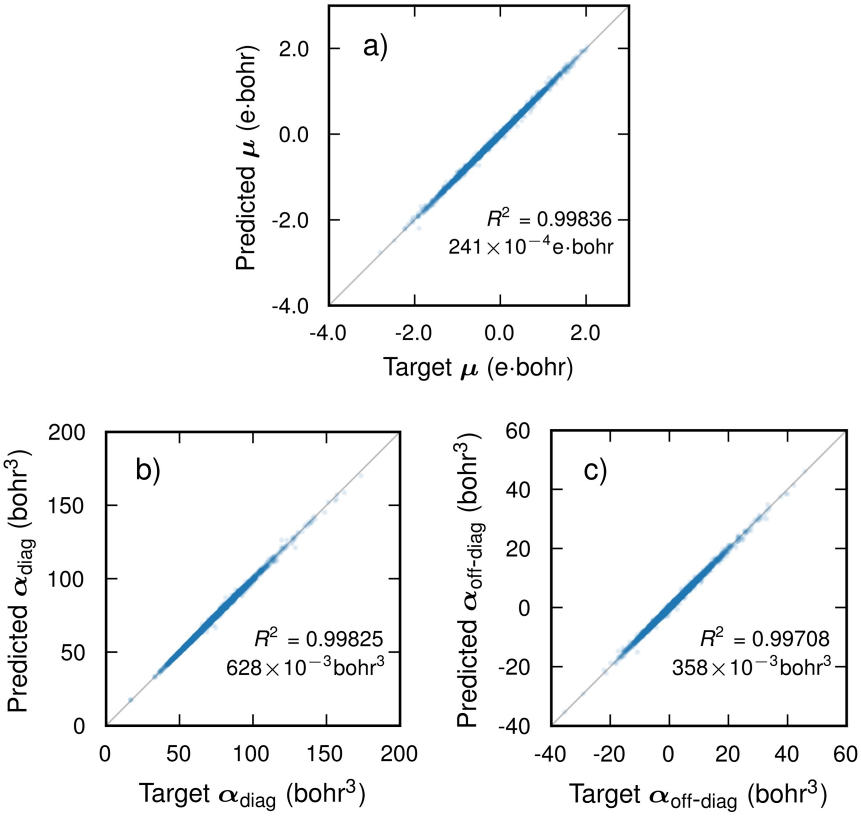

Figure S3: TNEP predictions as compared to CCSD reference data of (a) dipole moment, (b) diagonal elements of the polarizability, and (c) off-diagonal elements of the polarizability for the validation set of the QM7b set.

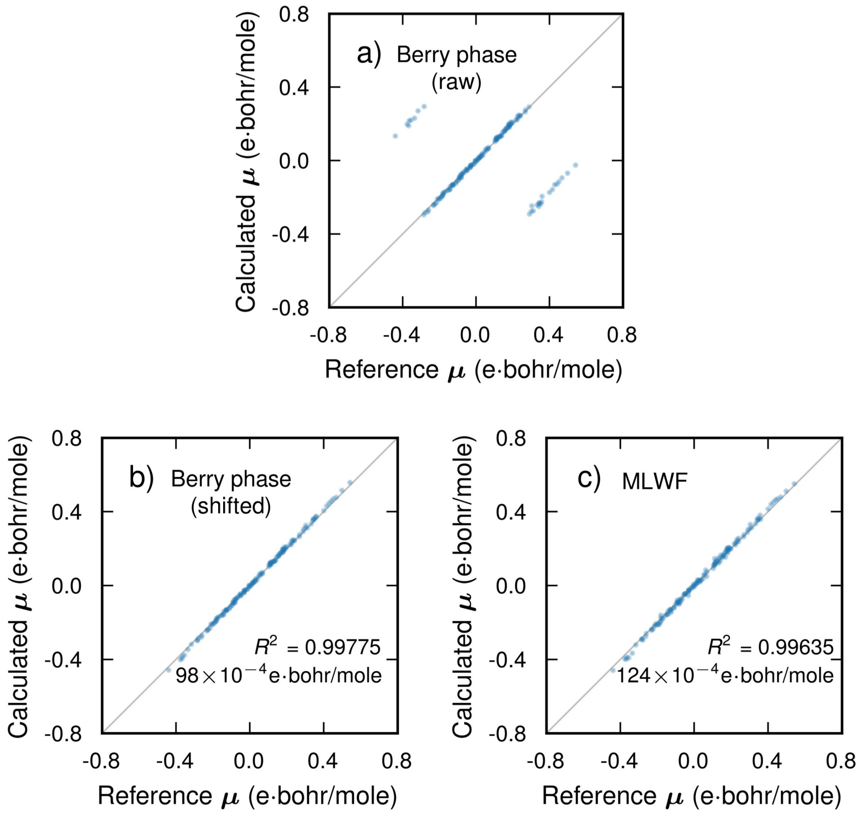

Figure S4: Dipole moments per molecule calculated in this work compared to reference data[1,2] for 50 liquid water structures that were evaluated in this work.

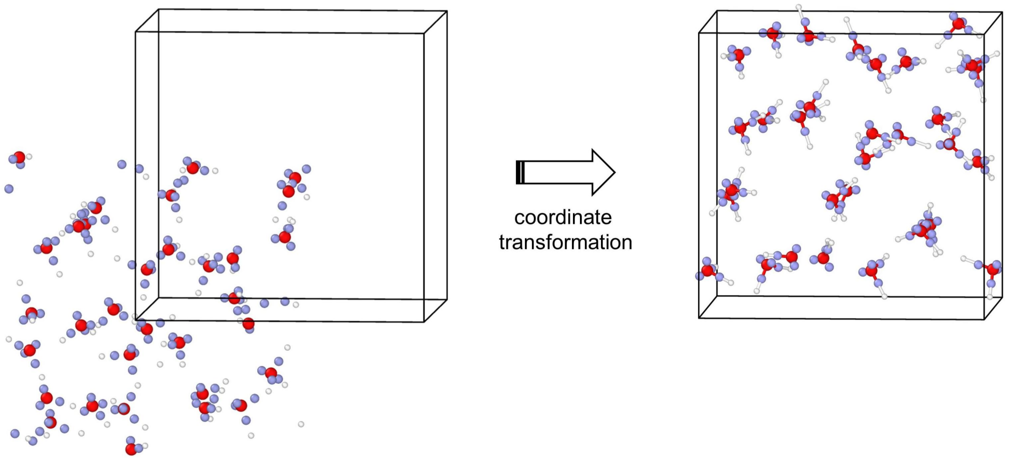

Figure S5: Schematic plot of coordinate transformation of water molecules and their Wannier centers before calculating the total dipole moment. Red, white, and slate-blue balls represent oxygen atoms, hydrogen atoms, and Wannier centers, respectively.

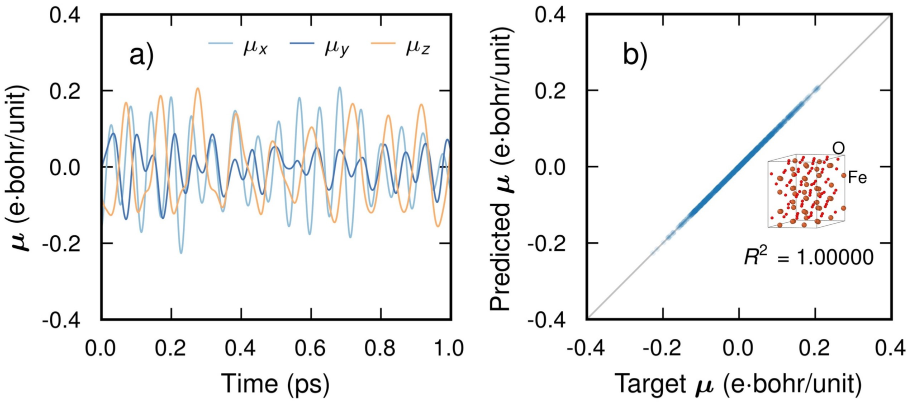

Figure S6: (a) Dipole moment per formula unit of α-Fe_2O_3 calculated by DFT as a function of simulation time. (b) The comparison between the TNEP predictions and DFT values of dipole moment for the validation data set of α-Fe_2O_3.

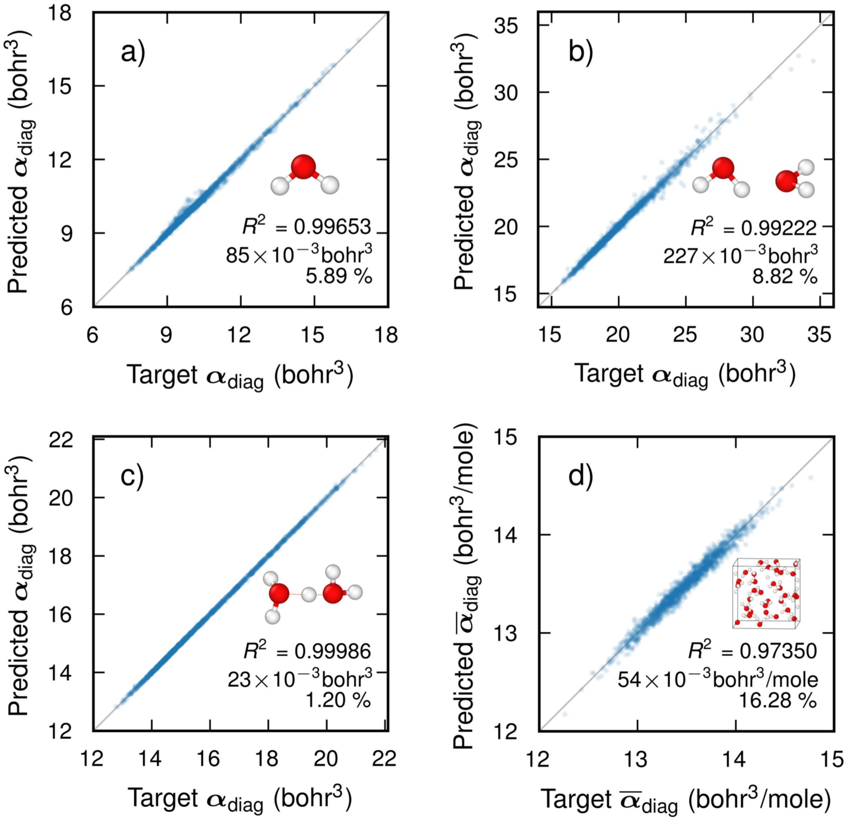

Figure S7: TNEP predicted diagonal polarizability as compared to *ab initio* data for the validation sets of (a) H_2O, (b) $(H_2O)_2$, (c) $H_5O_2{}^+$, and (d) liquid water. For liquid water we show the effective polarizability given by $\bar{\boldsymbol{\alpha}} = \boldsymbol{\chi}/\rho$, where ρ in the number volume density. R^2 scores, RMSEs, and RRMSEs are given in each subpanel. For liquid water the polarizability is divided given per water molecule.

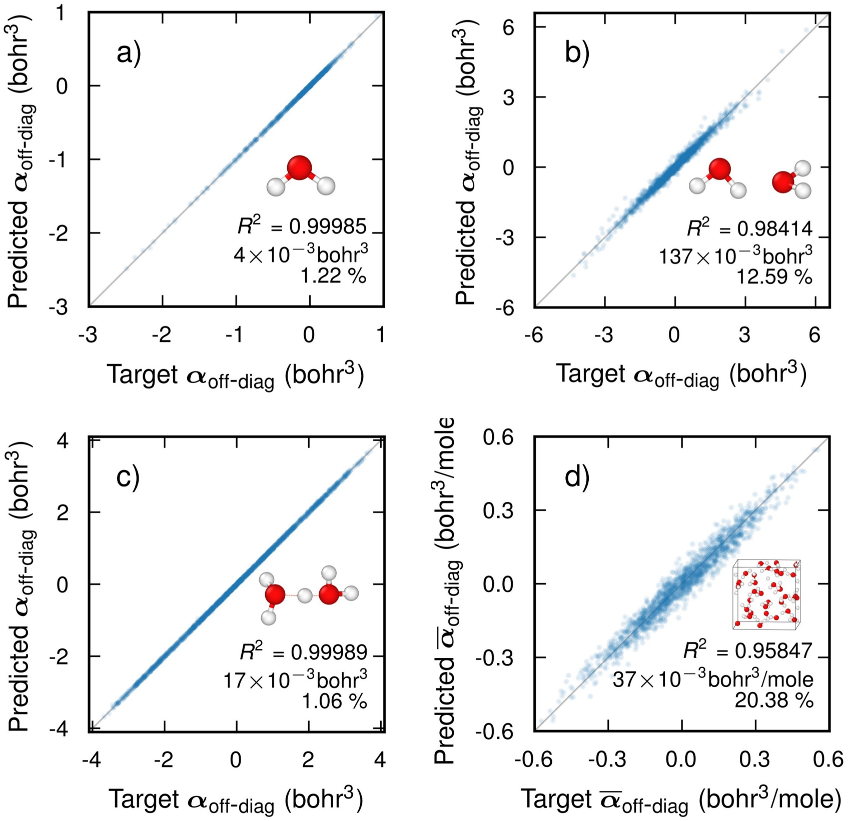

Figure S8: TNEP predicted off-diagonal polarizability compared to the *ab initio* values for the validation sets of (a) H_2O, (b) $(H_2O)_2$, (c) $H_5O_2{}^+$, and (d) liquid water. For liquid water we show the effective polarizability given by $\bar{\boldsymbol{\alpha}} = \boldsymbol{\chi}/\rho$, where ρ in the number volume density. R^2 scores, RMSEs, and RRMSEs are given in each subpanel. For liquid water the polarizability is given per water molecule.

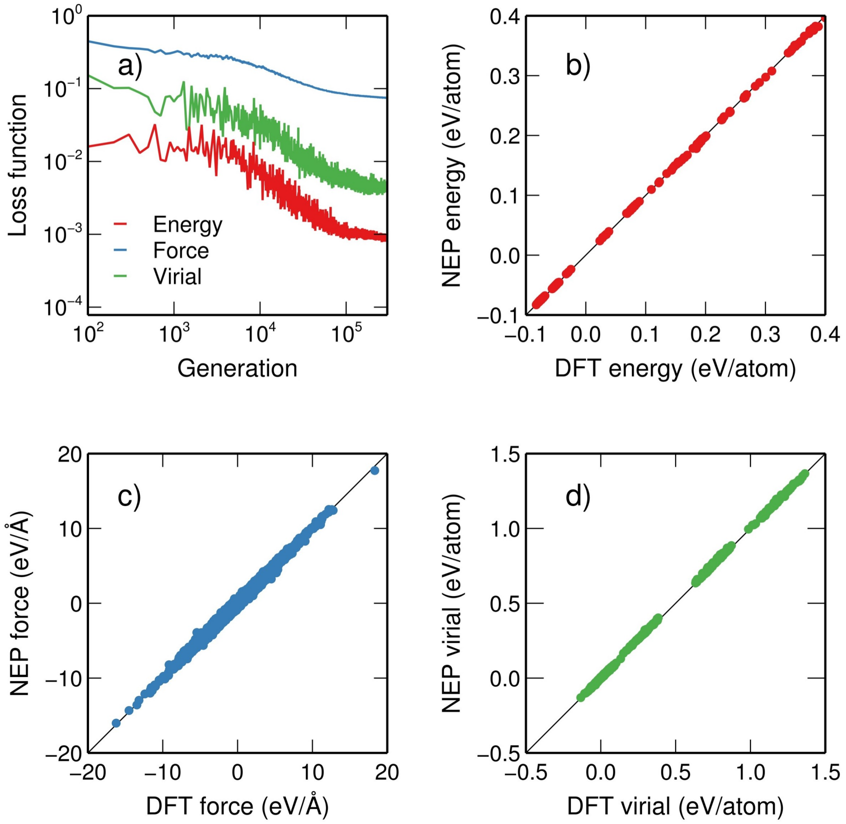

Figure S9: NEP model for the PES of liquid water. (a) RMSEs of energy, force, and virial for the validation set as a function of the number of generations. (b–d) Comparison between NEP predictions and DFT reference values of energies, forces, and virials for the validation set of liquid water.

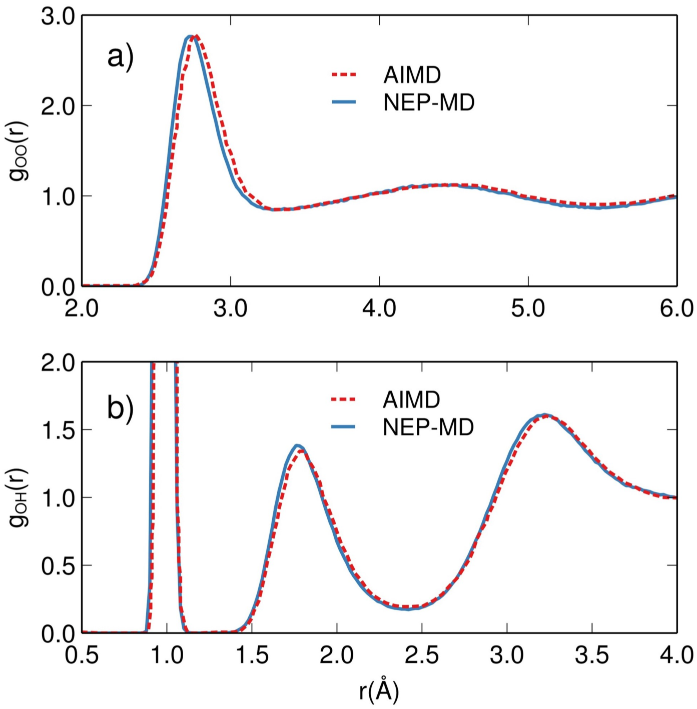

Figure S10: Validation of NEP model for the PES of liquid water. RDFs for (a) O–O and (b) O–H pairs extracted from MD simulations based on the NEP PES model constructed in this work and AIMD simulations[20] at 330 K and 1 bar.

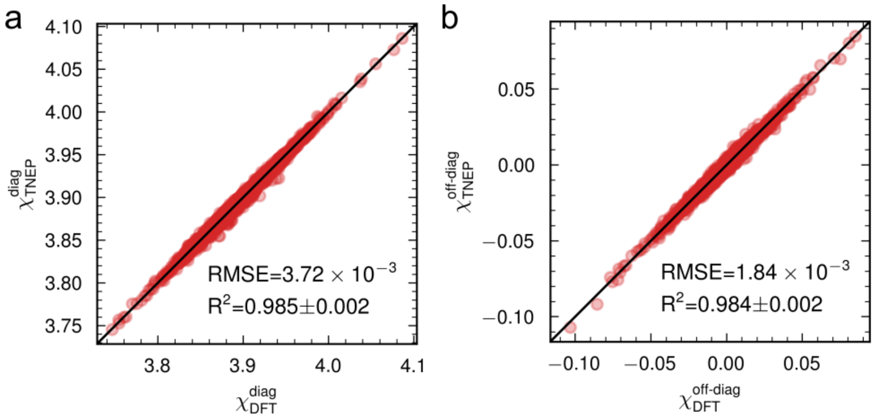

Figure S11: Comparison between TNEP predictions and DFT reference values for the (a) diagonal and (b) off-diagonal elements of the susceptibility $\boldsymbol{\chi}$ of $BaZrO_3$. The R^2 scores and RMSEs represent the means from five-fold hold-one-out cross-validation.

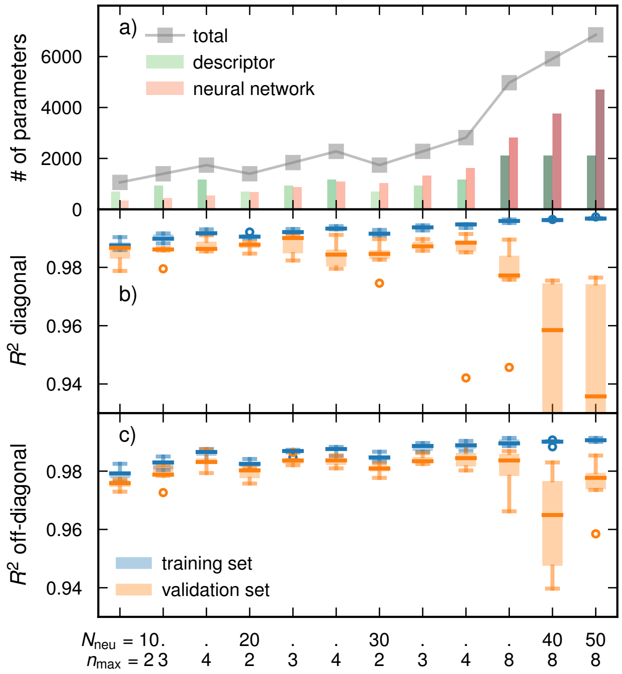

Figure S12: Impact of neural network architecture on model performance for TNEP models for the susceptibility of $\boldsymbol{\chi}$ of $BaZrO_3$. (a) The number of model parameters split by descriptor and neural network. (b, c) Box plots of R^2 for the (b) diagonal and (c) off-diagonal components of $\boldsymbol{\chi}$; outliers are shown by small open circles. N_{neu} indicates the number of neurons in the hidden layer of the neural network. n_{max} specifies the number of basis functions used to construct the radial ($n^{\mathrm{R}}_{\mathrm{max}}$) and angular ($n^{\mathrm{A}}_{\mathrm{max}}$) descriptors; see Ref. 34 for the full expressions for the network and the descriptors. Here, we use $n_{\mathrm{max}} = n^{\mathrm{R}}_{\mathrm{max}} = n^{\mathrm{A}}_{\mathrm{max}}$. Training was carried out using training sets generated by k-fold splitting of the 940 structures available. The comparison demonstrates that viable models can be obtained for a wide range of parameters, and that even small models with as few as 1500 or so parameters can yield very good results. Yet fine-tuning of the architecture (and the regularization parameters, see Fig. S13) allows one to optimize model performance.

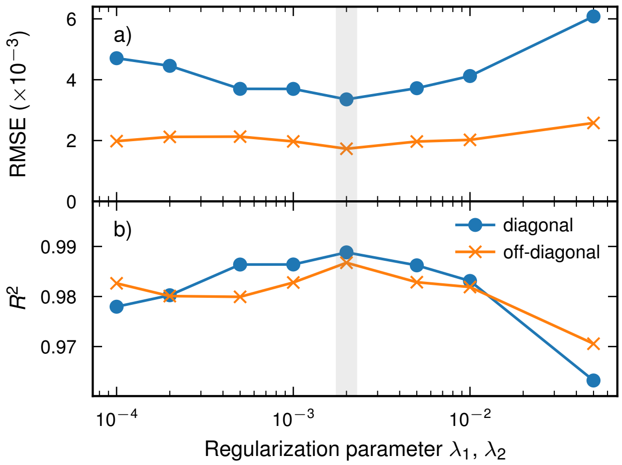

Figure S13: Hyperparameter tuning for TNEP models for the susceptibility of $\boldsymbol{\chi}$ of $BaZrO_3$. Variation of (a) RMSE and (b) R^2 for a validation set comprising 140 structures with the hyperparameters $\lambda_1 = \lambda_2$ that set the strength of the L_1 and L_2-norm regularization terms in the loss function. The value of $\lambda_1 = \lambda_2 = 2 \times 10^{-3}$ used for building the models in Fig. 6 is indicated by gray bars. Training was carried out using a set comprising 800 structures. The neural network contained $N_{\mathrm{neu}} = 20$ neurons and the radial and angular descriptors were constructed using $n_{\max} = 4$ (see Fig. S12).

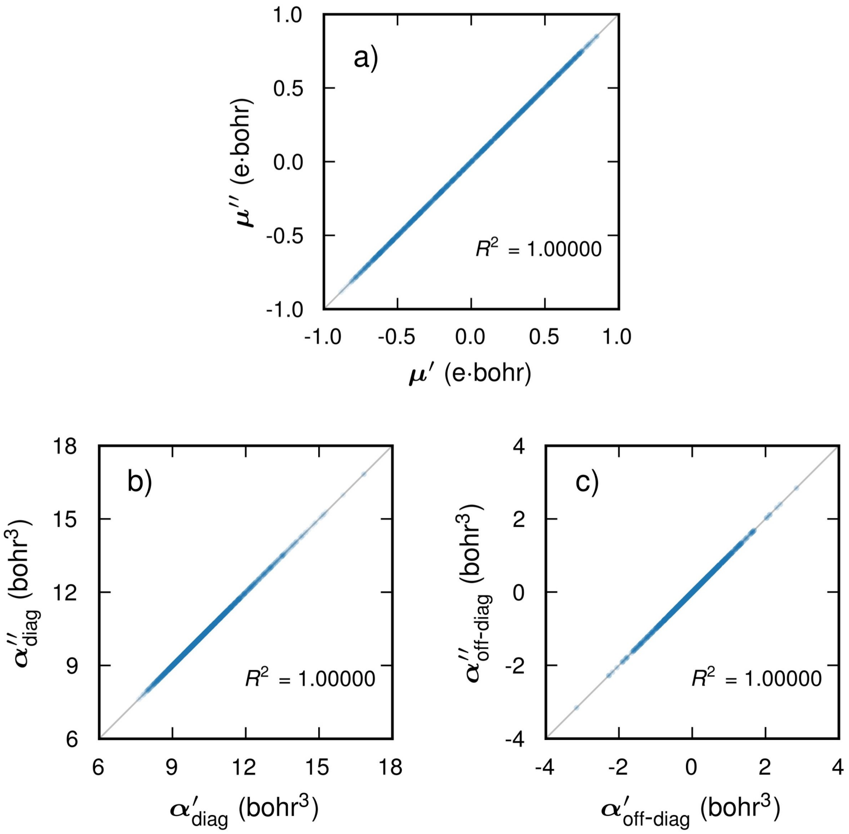

Figure S14: Demonstration of rotational invariance of TNEP predictions. The comparison shows the prediction for unrotated structures (with superscript $''$) vs predictions for rotated structures (with superscript $'$) of (a) dipole moment, (b) diagonal elements of the polarizability, and (c) off-diagonal elements of the polarizability for the validation set of monomeric H_2O.

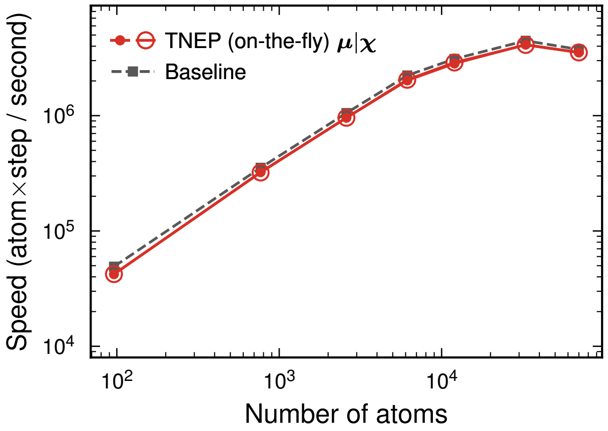

Figure S15: Scaling benchmark demonstrating cost of evaluating dipoles or polarizabilities/susceptibilities on-the-fly during MD simulations. The speed is compared to baseline MD simulations without predictions of tensorial properties, i.e., only sampling the PES. The system under study is bulk water at 300 K in the NVT ensemble, simulated with a timestep of 0.5 fs. The benchmark was run on a heterogeneous server with a Intel Core i7-4770K CPU and a RTX 3080Ti GPU with 12 GB VRAM.

Table S1: Hyperparameters used in training of TNEP models for the dipole moment ($\boldsymbol{\mu}$) for H_2O, $(H_2O)_2$, $H_5O_2{}^+$, liquid water, organic molecules (QM7B set), and α-Fe_2O_3. Here, r_c^{R} (r_c^{A}) is the cutoff radius for the radial (angular) components of the descriptor, $n_{\mathrm{max}}^{\mathrm{R}}$ ($n_{\mathrm{max}}^{\mathrm{A}}$) is the Chebyshev polynomial expansion order for the radial (angular) components, l_{max}^{3b} (l_{max}^{4b}, l_{max}^{5b}) is the Legendre polynomial expansion order for the three-body (four-body, five-body) terms angular components, $N_{\mathrm{bas}}^{\mathrm{R}}$ ($N_{\mathrm{bas}}^{\mathrm{A}}$) is the number of basis functions that are used to build the radial (angular) descriptor functions, N_{neu} is the number of neurons in the hidden layer of the neural network, λ_1 (λ_2) is the $\mathcal{L}_1$ ($\mathcal{L}_2$) regularization parameter, N_{pop} is the population size in the natural evolution strategy algorithm, N_{bat} is the size of each batch used during training, and N_{gen} is the maximum number of generations to be evolved.

Parameter	H_2O	$(H_2O)_2$	$H_5O_2{}^+$	liquid water	QM7B set	α-Fe_2O_3
r_c^{R} (Å)	6	6	6	6	6	6
r_c^{A} (Å)	4	4	4	4	4	4
$n_{\mathrm{max}}^{\mathrm{R}}$	6	6	6	6	6	6
$n_{\mathrm{max}}^{\mathrm{A}}$	6	6	6	6	6	6
l_{max}^{3b}	4	4	4	4	4	4
l_{max}^{4b}	2	2	2	2	2	2
l_{max}^{5b}	1	1	1	1	1	1
$N_{\mathrm{bas}}^{\mathrm{R}}$	10	10	10	10	10	10
$N_{\mathrm{bas}}^{\mathrm{A}}$	10	10	10	10	10	10
N_{neu}	10	10	10	10	30	10
λ_1	0.00005	0.0008	0.0012	0.0005	0.001	0.0001
λ_2	0.00005	0.0008	0.0012	0.0005	0.001	0.0001
N_{batch}	full-batch	full-batch	full-batch	full-batch	full-batch	full-batch
N_{pop}	80	80	80	80	80	80
N_{gen}	2×10^5	2×10^5	2×10^5	2×10^5	4×10^5	2×10^5

Table S2: Hyperparameters used in training of TNEP polarizability and susceptibility models for H_2O, $(H_2O)_2$, $H_5O_2{}^+$, liquid water, and $BaZrO_3$. Compared to the TNEP dipole model, an additional parameter λ_s should be set, which stands for the relative weight between the off-diagonal elements and diagonal elements of rank-2 tensors in the construction of loss functions.

Parameter	H_2O	$(H_2O)_2$	$H_5O_2{}^+$	liquid water	QM7B set	$BaZrO_3$
$r_c^{\rm R}$ (Å)	6	6	6	6	6	6
$r_c^{\rm A}$ (Å)	4	4	4	4	4	4
$n_{\rm max}^{\rm R}$	6	6	6	6	6	4
$n_{\rm max}^{\rm A}$	6	6	6	6	6	4
$l_{\rm max}^{3b}$	4	4	4	4	4	4
$l_{\rm max}^{4b}$	2	2	2	2	2	0
$l_{\rm max}^{5b}$	1	1	1	1	1	0
$N_{\rm bas}^{\rm R}$	10	10	10	10	10	12
$N_{\rm bas}^{\rm A}$	10	10	10	10	10	12
$N_{\rm neu}$	10	10	10	10	30	20
λ_1	0.008	0.02	0.002	0.001	0.03	-1 (adaptive adjustment)
λ_2	0.008	0.02	0.002	0.001	0.03	-1 (adaptive adjustment)
$N_{\rm batch}$	full-batch	full-batch	full-batch	full-batch	full-batch	full-batch
$N_{\rm pop}$	80	80	80	80	80	50
$N_{\rm gen}$	2×10^5	2×10^5	2×10^5	2×10^5	4×10^5	6×10^5
λ_s	10	1	1	1	1	1

Table S3: Hyperparameters used in training a NEP PES model for MD simulations of water.

Parameter	Liquid water
$r_c^{\rm R}$ (Å)	6
$r_c^{\rm A}$ (Å)	4
$n_{\rm max}^{\rm R}$	9
$n_{\rm max}^{\rm A}$	7
$l_{\rm max}^{3b}$	4
$l_{\rm max}^{4b}$	2
$l_{\rm max}^{5b}$	0
$N_{\rm bas}^{\rm R}$	9
$N_{\rm bas}^{\rm A}$	7
$N_{\rm neu}$	100
λ_1	-1 (adaptive adjustment)
λ_2	-1 (adaptive adjustment)
$N_{\rm batch}$	750
$N_{\rm pop}$	50
$N_{\rm gen}$	3×10^5

Table S4: Hyperparameters used in training a NEP models for the prediction of infrared spectra for $PTAF^-$.

Parameter	$PTAF^-$ PES	$PTAF^-$ $\boldsymbol{\mu}$
r_c^{R} (Å)	8	8
r_c^{A} (Å)	4	6
$n_{\mathrm{max}}^{\mathrm{R}}$	8	15
$n_{\mathrm{max}}^{\mathrm{A}}$	6	8
l_{max}^{3b}	4	4
l_{max}^{4b}	0	2
l_{max}^{5b}	0	0
$N_{\mathrm{bas}}^{\mathrm{R}}$	8	12
$N_{\mathrm{bas}}^{\mathrm{A}}$	8	12
N_{neu}	40	80
λ_1	0.1	-1 (adaptive adjustment)
λ_2	0.1	-1 (adaptive adjustment)
λ_e	1	1
λ_f	3	1
λ_v	0	0.1
N_{batch}	1×10^5	5×10^5
N_{pop}	50	50
N_{gen}	2×10^5	5×10^5

Table S5: Validation data for dipole and polarizability of water. RRMSEs (unitless) for $\boldsymbol{\mu}$ and $\boldsymbol{\alpha}$ for the validation sets using NEP, T-EANN and SA-GPR rank-1 tensor models.

	$\boldsymbol{\mu}$			$\boldsymbol{\alpha}$		
System	TNEP	T-EANN	SA-GPR	TNEP	T-EANN	SA-GPR
H_2O	0.069%	0.020%	0.023%	0.991%	0.020%	0.024%
$(H_2O)_2$	1.681%	6.600%	3.866%	1.762%	4.200%	1.258%
$H_5O_2{}^+$	0.371%	1.300%	0.130%	0.246%	0.300%	0.080%
liquid water	0.852%	16.000%	0.544%	0.680%	2.200%	0.329%

Supplemental References

[1] TENSOAP, accessed: 12-08-2023. https://github.com/dilkins/TENSOAP.

[2] Andrea Grisafi, David M. Wilkins, Gábor Csányi, and Michele Ceriotti. Symmetry-adapted machine learning for tensorial properties of atomistic systems. *Phys. Rev. Lett.*, 120(3):036002, 2018. doi: 10.1103/PhysRevLett.120.036002.

[3] Hendrik J. Monkhorst. Calculation of properties with the coupled-cluster method. *Int. J. Quantum Chem.*, 12(S11):421–432, 1977. doi: https://doi.org/10.1002/qua.560120850. URL https://onlinelibrary.wiley.com/doi/abs/10.1002/qua.560120850.

[4] Henrik Koch and Poul Jørgensen. Coupled cluster response functions. *J. Chem. Phys.*, 93(5): 3333–3344, 1990. doi: 10.1063/1.458814. URL https://doi.org/10.1063/1.458814.

[5] David E. Woon and Thom H. Dunning. Gaussian basis sets for use in correlated molecular calculations. iv. calculation of static electrical response properties. *J. Chem. Phys.*, 100(4):2975–2988, 1994. doi: 10.1063/1.466439. URL https://doi.org/10.1063/1.466439.

[6] Thom H. Dunning. Gaussian basis sets for use in correlated molecular calculations. i. the atoms boron through neon and hydrogen. *J. Chem. Phys.*, 90(2):1007–1023, 1989. ISSN 0021-9606. doi: 10.1063/1.456153. URL https://aip.scitation.org/doi/10.1063/1.456153.

[7] John P. Perdew, Kieron Burke, and Matthias Ernzerhof. Generalized gradient approximation made simple. *Phys. Rev. Lett.*, 77:3865–3868, Oct 1996. doi: 10.1103/PhysRevLett.77.3865. URL https://link.aps.org/doi/10.1103/PhysRevLett.77.3865.

[8] David Vanderbilt. Soft self-consistent pseudopotentials in a generalized eigenvalue formalism. *Phys. Rev. B*, 41:7892–7895, Apr 1990. doi: 10.1103/PhysRevB.41.7892. URL https://link.aps.org/doi/10.1103/PhysRevB.41.7892.

[9] Nicola A. Spaldin. A beginner's guide to the modern theory of polarization. *J. Solid State Chem.*, 195:2–10, 2012. ISSN 0022-4596. doi: https://doi.org/10.1016/j.jssc.2012.05.010. URL https://www.sciencedirect.com/science/article/pii/S0022459612003234.

[10] Aravind Krishnamoorthy, Ken-ichi Nomura, Nitish Baradwaj, Kohei Shimamura, Pankaj Rajak, Ankit Mishra, Shogo Fukushima, Fuyuki Shimojo, Rajiv Kalia, Aiichiro Nakano, and Priya Vashishta. Dielectric constant of liquid water determined with neural network quantum molecular dynamics. *Phys. Rev. Lett.*, 126:216403, May 2021. doi: 10.1103/PhysRevLett.126.216403. URL https://link.aps.org/doi/10.1103/PhysRevLett.126.216403.

[11] Thomas D. Kühne, Marcella Iannuzzi, Mauro Del Ben, Vladimir V. Rybkin, Patrick Seewald, Frederick Stein, Teodoro Laino, Rustam Z. Khaliullin, Ole Schütt, Florian Schiffmann, Dorothea Golze, Jan Wilhelm, Sergey Chulkov, Mohammad Hossein Bani-Hashemian, Valéry Weber, Urban Borštnik, Mathieu Taillefumier, Alice Shoshana Jakobovits, Alfio Lazzaro, Hans Pabst, Tiziano Müller, Robert Schade, Manuel Guidon, Samuel Andermatt, Nico Holmberg, Gregory K. Schenter, Anna Hehn, Augustin Bussy, Fabian Belleflamme, Gloria Tabacchi, Andreas Glöß, Michael Lass, Iain Bethune, Christopher J. Mundy, Christian Plessl, Matt Watkins, Joost VandeVondele, Matthias Krack, and Jürg Hutter. CP2K: An electronic structure and molecular dynamics software package - Quickstep: Efficient and accurate electronic structure calculations. *J. Chem. Phys.*, 152(19):194103, 05 2020. doi: 10.1063/5.0007045.

[12] Joost VandeVondele and Jürg Hutter. Gaussian basis sets for accurate calculations on molecular systems in gas and condensed phases. *J. Chem. Phys.*, 127(11), 09 2007. ISSN 0021-9606. doi: 10.1063/1.2770708. URL https://doi.org/10.1063/1.2770708. 114105.

[13] Stefan Grimme, Jens Antony, Stephan Ehrlich, and Helge Krieg. A consistent and accurate ab initio parametrization of density functional dispersion correction (DFT-D) for the 94 elements H-Pu. *J. Chem. Phys.*, 132(15), 04 2010. ISSN 0021-9606. doi: 10.1063/1.3382344. URL https://doi.org/10.1063/1.3382344. 154104.

[14] Nicola Marzari, Arash A. Mostofi, Jonathan R. Yates, Ivo Souza, and David Vanderbilt. Maximally localized wannier functions: Theory and applications. *Rev. Mod. Phys.*, 84:1419–1475, Oct 2012. doi: 10.1103/RevModPhys.84.1419. URL `https://link.aps.org/doi/10.1103/RevModPhys.84.1419`.

[15] Zifan Ye, Francois Gygi, and Giulia Galli. Raman Spectra of Electrified Si–Water Interfaces: First-Principles Simulations. *The Journal of Physical Chemistry Letters*, 15(1):51–58, January 2024. doi: 10.1021/acs.jpclett.3c03122.

[16] Ke Xu, Yongchao Hao, Ting Liang, Penghua Ying, Jianbin Xu, Jianyang Wu, and Zheyong Fan. Accurate prediction of heat conductivity of water by a neuroevolution potential. *J. Chem. Phys.*, 158(20), 05 2023. ISSN 0021-9606. doi: 10.1063/5.0147039. URL `https://doi.org/10.1063/5.0147039`.

[17] Linfeng Zhang, Han Wang, Roberto Car, and Weinan E. Phase diagram of a deep potential water model. *Phys. Rev. Lett.*, 126:236001, Jun 2021. doi: 10.1103/PhysRevLett.126.236001. URL `https://link.aps.org/doi/10.1103/PhysRevLett.126.236001`.

[18] Kerwin Hui and Jeng-Da Chai. Scan-based hybrid and double-hybrid density functionals from models without fitted parameters. *J. Chem. Phys.*, 144(4):044114, 2016. doi: 10.1063/1.4940734. URL `https://doi.org/10.1063/1.4940734`.

[19] P. E. Blöchl. Projector augmented-wave method. *Phys. Rev. B*, 50:17953–17979, Dec 1994. doi: 10.1103/PhysRevB.50.17953. URL `https://link.aps.org/doi/10.1103/PhysRevB.50.17953`.

[20] Mohan Chen, Hsin-Yu Ko, Richard C. Remsing, Marcos F. Calegari Andrade, Biswajit Santra, Zhaoru Sun, Annabella Selloni, Roberto Car, Michael L. Klein, John P. Perdew, and Xifan Wu. Ab initio theory and modeling of water. *Proc. Natl. Acad. Sci. U.S.A.*, 114(41):10846–10851, 2017. URL `https://www.pnas.org/doi/abs/10.1073/pnas.1712499114`.

[21] Matthias Rupp, Alexandre Tkatchenko, Klaus-Robert Müller, and O. Anatole von Lilienfeld. Fast and accurate modeling of molecular atomization energies with machine learning. *Phys. Rev. Lett.*, 108:058301, Jan 2012. doi: 10.1103/PhysRevLett.108.058301. URL `https://link.aps.org/doi/10.1103/PhysRevLett.108.058301`.

[22] Yang Yang, Ka Un Lao, David M. Wilkins, Andrea Grisafi, Michele Ceriotti, and Robert A. DiStasio. Quantum mechanical static dipole polarizabilities in the qm7b and alphaml showcase databases. *Sci. Data*, 6(1):152, 2019. doi: 10.1038/s41597-019-0157-8. URL `https://doi.org/10.1038/s41597-019-0157-8`.

[23] Yassine El Mendili, Jean-François Bardeau, Nirina Randrianantoandro, Fabien Grasset, and Jean-Marc Greneche. Insights into the mechanism related to the phase transition from γ-Fe_2O_3 to α-Fe_2O_3 nanoparticles induced by thermal treatment and laser irradiation. *J. Phys. Chem. C*, 116(44):23785–23792, 2012.

[24] Gerald Lippert, Jörg Hutter, and Michele Parrinello. A hybrid gaussian and plane wave density functional scheme. *Mol. Phys.*, 92(3):477–488, 1997.

[25] Joost VandeVondele, Matthias Krack, Fawzi Mohamed, Michele Parrinello, Thomas Chassaing, and Jürg Hutter. Quickstep: Fast and accurate density functional calculations using a mixed gaussian and plane waves approach. *Comput. Phys. Commun.*, 167(2):103–128, 2005. ISSN 0010-4655. doi: https://doi.org/10.1016/j.cpc.2004.12.014. URL `https://www.sciencedirect.com/science/article/pii/S0010465505000615`.

[26] Glenn J. Martyna, Michael L. Klein, and Mark Tuckerman. Nosé–Hoover chains: The canonical ensemble via continuous dynamics. *J. Chem. Phys.*, 97(4):2635–2643, 08 1992. ISSN 0021-9606. doi: 10.1063/1.463940. URL `https://doi.org/10.1063/1.463940`.

[27] Erik Fransson, Petter Rosander, Paul Erhart, and Göran Wahnström. Understanding correlations in $BaZrO_3$: Structure and dynamics on the nano-scale. *Chemistry of Materials*, 36:514–523, 7 2024. doi: 10.1021/acs.chemmater.3c02548.

[28] G. Kresse and D. Joubert. From ultrasoft pseudopotentials to the projector augmented-wave method. *Physical Review B*, 59(3):1758–1775, 1999. doi: 10.1103/PhysRevB.59.1758.

[29] G. Kresse and J. Hafner. Ab initio molecular dynamics for liquid metals. *Physical Review B*, 47: 558–561, Jan 1993. doi: 10.1103/PhysRevB.47.558.

[30] G. Kresse and J. Furthmüller. Efficiency of ab-initio total energy calculations for metals and semiconductors using a plane-wave basis set. *Computational Materials Science*, 6(1):15–50, 1996. doi: 10.1016/0927-0256(96)00008-0.

[31] M. Dion, H. Rydberg, E. Schröder, D. C. Langreth, and B. I. Lundqvist. Van der waals density functional for general geometries. *Phys. Rev. Lett.*, 92:246401, 2004. doi: 10.1103/PhysRevLett.92.246401.

[32] Kristian Berland and Per Hyldgaard. Exchange functional that tests the robustness of the plasmon description of the van der waals density functional. *Physical Review B*, 89:035412, Jan 2014. doi: 10.1103/PhysRevB.89.035412.

[33] Kaoru Urano and Masayoshi Inoue. Clausius–Mossotti formula for anisotropic dielectrics. *J. Chem. Phys*, 66(2):791–794, 08 2008. doi: 10.1063/1.433957.

[34] Zheyong Fan, Yanzhou Wang, Penghua Ying, Keke Song, Junjie Wang, Yong Wang, Zezhu Zeng, Ke Xu, Eric Lindgren, J. Magnus Rahm, Alexander J. Gabourie, Jiahui Liu, Haikuan Dong, Jianyang Wu, Yue Chen, Zheng Zhong, Jian Sun, Paul Erhart, Yanjing Su, and Tapio Ala-Nissila. Gpumd: A package for constructing accurate machine-learned potentials and performing highly efficient atomistic simulations. *J. Chem. Phys.*, 157(11):114801, 2022. ISSN 0021-9606. doi: 10.1063/5.0106617.
